# Cytotoxic unsaturated electrophilic compounds commonly target the ubiquitin proteasome system

**DOI:** 10.1038/s41598-019-46168-x

**Published:** 2019-07-08

**Authors:** Karthik Selvaraju, Arjan Mofers, Paola Pellegrini, Johannes Salomonsson, Alexandra Ahlner, Vivian Morad, Ellin-Kristina Hillert, Belen Espinosa, Elias S. J. Arnér, Lasse Jensen, Jonas Malmström, Maria V. Turkina, Padraig D’Arcy, Michael A. Walters, Maria Sunnerhagen, Stig Linder

**Affiliations:** 1 Department of Medical and Health Sciences, Linköping University, SE-58183 Linköping, Sweden; 2 Department of Physics, Chemistry and Biology, Linköping University, SE-58183 Linköping, Sweden; 3 Department of Oncology-Pathology, Karolinska Institutet, SE-17176 Stockholm, Sweden; 4 Division of Biochemistry, Department of Medical Biochemistry and Biophysics, Karolinska Institutet, SE-17177 Stockholm, Sweden; 5 Recipharm AB, Box CB11303, SE-101 32 Stockholm, Sweden; 6 Department of Clinical and Experimental Medicine SE-58185 Linköping University, Linköping, Sweden; 7 Department of Medicinal Chemistry, Institute for Therapeutics Discovery and Development, University of Minnesota, Minnesota, United States

**Keywords:** Drug development, Deubiquitylating enzymes

## Abstract

A large number of natural products have been advocated as anticancer agents. Many of these compounds contain functional groups characterized by chemical reactivity. It is not clear whether distinct mechanisms of action can be attributed to such compounds. We used a chemical library screening approach to demonstrate that a substantial fraction (~20%) of cytotoxic synthetic compounds containing Michael acceptor groups inhibit proteasome substrate processing and induce a cellular response characteristic of proteasome inhibition. Biochemical and structural analyses showed binding to and inhibition of proteasome-associated cysteine deubiquitinases, in particular ubiquitin specific peptidase 14 (USP14). The results suggested that compounds bind to a crevice close to the USP14 active site with modest affinity, followed by covalent binding. A subset of compounds was identified where cell death induction was closely associated with proteasome inhibition and that showed significant antineoplastic activity in a zebrafish embryo model. These findings suggest that proteasome inhibition is a relatively common mode of action by cytotoxic compounds containing Michael acceptor groups and help to explain previous reports on the antineoplastic effects of natural products containing such functional groups.

## Introduction

Natural products have a privileged position in drug discovery^1^. The scaffold diversity of natural products leads to interactions with different cellular targets^2^. However, natural products frequently contain unwanted structural elements leading to potentially high levels of wide-spread reactivity and the usefulness of natural products as leads for hit-to-lead drug discovery has been discussed in recent reviews^3,4^. Natural products frequently contain Pan Assay INterference compoundS (PAINS) motifs^4^, the awareness of which has increased in the scientific community^5,6^. The PAINS substructure filters have been accepted in the medicinal chemistry community as a useful and straightforward method of flagging potentially problematic active compounds in high-throughput screening campaigns^5–8^. One seventh of all natural products contain Michael acceptors^3^ and depend on these structures for activity^9–11^. Compounds containing reactive groups should not be used as chemical probes^12,13^. Many natural products do, however, show antineoplastic activity in animal models and are therefore likely to induce cellular responses that are not generally toxic. One such response is inhibition of the ubiquitin-proteasome system (UPS), observed by natural products such as withaferin A^14,15^, celastrol^16^, piperlongumine^17,18^, gambogic acid^19^ and curcumin^20^, all containing Michael acceptor groups. In addition to inhibition of the proteasome, natural products containing α,β-unsaturated carbonyl functionalities are known to inhibit thioredoxin reductase 1 (TrxR1), a selenoprotein with an exceptionally reactive nucleophile that is a preferred target of electrophilic compounds. TrxR1 is among the most susceptible enzyme inhibited by electrophilic compounds having anticancer activities^21,22^. Specific targeting of TrxR1 has recently been demonstrated to have antineoplastic effects *in vitro* and *in vivo*^23^

The proteasome is the major protein degradation system in eukaryotic cells and consists of a degradation complex in the form of the 26S proteasome and a system of enzymes that tag unwanted proteins with the 8.5 kDa protein ubiquitin for subsequent degradation^24,25^. Protein ubiquitination is a reversible process, deubiquitinases (DUBs) can remove ubiquitin from substrates by cleaving the isopeptide bond between the C terminus of ubiquitin and a lysine residue on a target protein^24,26^. The majority of different DUBs contain functional cysteines^27^ and are expected to be highly druggable. In humans, three DUBs are associated with the 19S regulatory particle of the proteasome, two of which (UCHL5/Uch37 and USP14/Ubp6) are cysteine proteases. A number of α,β-unsaturated compounds have been described to inhibit proteasome-associated DUBs, including the synthetic compounds b-AP15^28^ and RA-9^29^ and the curcumin analogue AC17^30^ (Supplementary Fig. 1a**)**.

Cancer is a disease characterized by the occurrence of a plethora of genetic and epigenetic alterations, clonal heterogeneity and plasticity^31^. The clinical efficacy of drugs that target mutant enzymes and receptors is hampered by the rapid development of resistant clones resulting in treatment failure^32,33^. Compounds with mechanisms relying on polypharmacology for their antineoplastic activity may therefore be beneficial, although potential harmful side effects must be carefully considered (for a discussion, see^34^). Whether natural products and other compounds that contain reactive elements induce a defined mechanism of action is, however, unclear. Considering the reports in the literature that various types of Michael acceptors possessing α,β-unsaturated carbonyl groups inhibit the UPS we here used a chemical biology approach to examine whether this type of mechanism is common for α,β-unsaturated compounds and whether compounds can be identified with mechanisms of action associated with proteasome inhibition.

## Results

### Selection of α,β-unsaturated compounds

**Figure 1:**
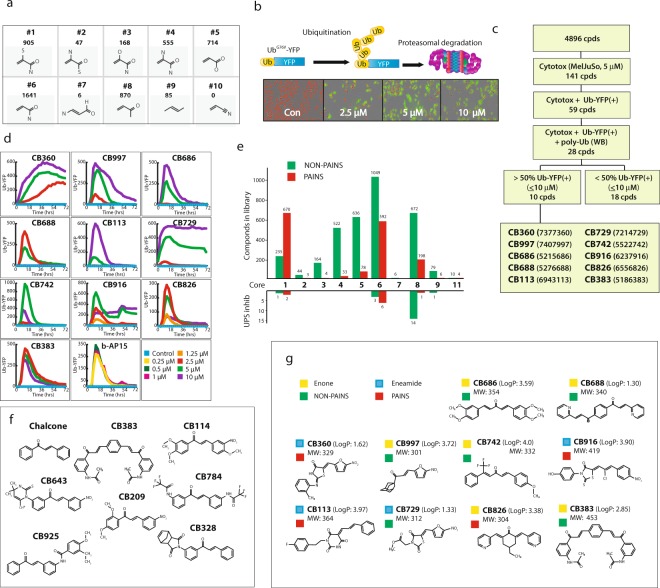
Screening of α,β-unsaturated compounds for cytotoxicity and inhibition of proteasome processing. (**a**) Substructures (cores) in the ChemBridge library of 5005 α,β-unsaturated compounds and number of compounds containing that substructure in the library. The core structures were uniquely assigned in order of molecular weight with the highest molecular weight being assigned first (**1–10**). Core **10** has no compounds in it because these were already assigned to other core structures. Core **11** (not shown) represents compounds unassigned to any core structure. (**b**) The ubiquitin^G76V^-YFP reporter system used in this study^69^. Shown is the accumulation of YFP in MelJuSo cells exposed to hit compound **CB360**. (**c**) Screening strategy used. Of the 5005 α,β-unsaturated compounds in the cLogP range 1–4 that were selected, 109 were not soluble in DMSO and were not used. Screening was performed using MelJuSo melanoma cells expressing a Nuclear Red marker and also the ubiquitin^G76V^-YFP reporter for determination of cell number and proteasome function. Fluorescence was recorded in an IncuCyte^®^ instrument. A total of 141 compounds were scored as cytotoxic and 59 to induce yellow color. Western blotting confirmed polyubiquitin accumulation in MelJuSo cells by 28 compounds, 10 of which were selected for further studies based on strongest ubiquitin^G76V^-YFP reporter activity. (**d**) Kinetics of induction of YFP-fluorescence by 10 hit compounds and by the positive control b-AP15^28^. Maintained fluorescence in cells over the course of the recordings of some of the hits is likely due to colored compounds adhering to cells. (**e**) Representation of the frequency of different classes of compounds in the screening library and in the set of proteasome inhibitors. The number of PAINS and NON-PAINS according to core (see **a**) is shown. PAINS were flagged using the software at FAFDrugs4 (http://fafdrugs3.mti.univ-paris-diderot.fr/). (**f**) Structure of chalcone compounds in the set of 28 hit compounds. (**g**) Structure of the 10 hit compounds studied in detail. cLogPs (ChemBridge) and molecular weights are indicated as well as classification as enones/eneamides and PAINS. Data base searches (PubChem CID) showed that these compounds have generally not been reported as “universal hits” in *in vitro* screens (of >2000 bioassay results presented on PubChem for these 28 compounds, <1% showed positivity in *in vitro* assays). Furthermore, a significant selection for hydrophobic compounds was not observed (see^68^).

A library of 5005 synthetic compounds containing α,β-unsaturated groups with cLogPs of 1–4 was selected. The library contained a number of different structural core elements, shown in Fig. 1a. We applied (Pan Assay INterfering compoundS) PAINS filters^5–8^ to see whether they would be of use in the triage of actives found in the screen of a library purposefully designed to contain electrophilic compounds. The PAINS filters were found to flag approximately one third of the compounds in the library (1588/5005 (31.7%)).

### Proteasome inhibition by α,β-unsaturated compounds

In order to monitor cytotoxicity and proteasome inhibition following exposure to different compounds we used a melanoma cell line (MelJuSo) that stably expresses both a NucLight Red fluorescent protein for assessment of cell number and a proteasome-degradable reporter protein (Ub^G76V^-YFP)^35^ (Fig. 1b). Of the 5005 compounds in library, 4896 were soluble in DMSO and were used for screening at a concentration of 5 μM. Using the NucLight Red as a read-out for viable MelJuSo cells, 141/4896 (2.9%) of the soluble compounds were scored as cytotoxic (Fig. 1c**)**. Of these 141 compounds, 59 produced yellow fluorescent signals indicating decreased proteasomal degradation of the Ub^G76V^-YFP reporter or, alternatively, cellular binding/uptake of yellow fluorescent compounds (Fig. 1c,d). The maximal number of YFP positive cells was generally observed at 12–14 h after addition of compounds, followed by a gradual decrease in YFP positivity due to cytotoxicity (Fig. 1d). Maintained yellow fluorescent signals were observed in some instances and appeared to be due to uptake or binding of fluorescent compounds to cells (see further below).

Considering the number of PAINS in the library and the generally acknowledged reactivity of the α,β-unsaturated carbonyl moiety it was important to perform a variety of different assays to confirm activity. We examined inhibition of proteasome activity using immunoblotting for polyubiquitinated substrates as a readout independent of compound autofluorescence (further described below). This narrowed the set to 28 active compounds preliminary classified as proteasome inhibitors. These compounds showed cytotoxic or cytostatic activity at 5 μM as evidenced from monitoring of cell proliferation **(**Fig. 1c, Supplementary Fig. 1c,d). Hit compounds were primarily eneamides (core #6, 9 compounds) and enones (core #8, 15 compounds) (Fig. 1e). Enones were enriched in the screen by a factor of ~3. Furthermore, only 6.7% (1/15) of the enone proteasome inhibitors were classified as PAINS. We also noticed that of 86 chalcones in the library, seven were proteasome inhibitors (Fig. 1f). Chalcones have been reported to show anticancer activity and to inhibit proteasome activity as well as other non-proteasome targets^36^. The ten most potent compounds were selected as “hit compounds” for further study, six enones and four eneamides. The structures of these compounds are shown in Fig. 1g. Three of the hit compounds have nitrofuran rings (**CB360**, **CB997** and **CB729**) and two compounds (**CB686** and **CB826)** have 1,5-diaryl-3-oxo-1,4,-pentadienyl pharmacophores similar to previously described DUB inhibitors^28,29,37^. Compound **CB916** shows some structural similarity to previously described IKKβ and SCF-Skp2 inhibitors^35,38^. Four of ten compounds were flagged as PAINS (red boxes in Fig. 1g).

### Hit compounds induce blocking of proteasomal processing

**Figure 2:**
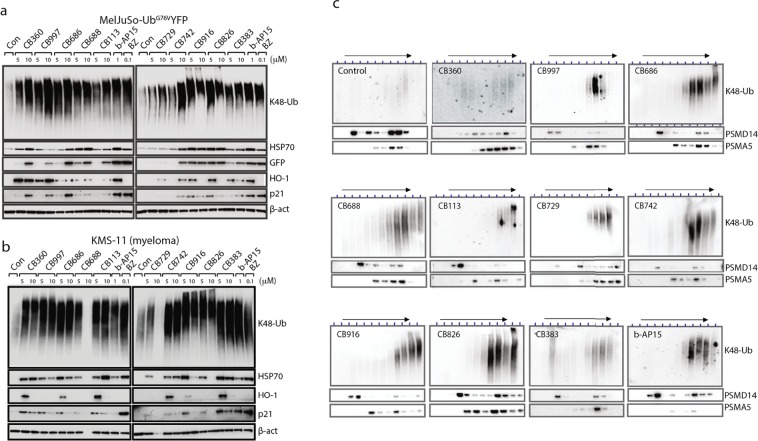
Proteasome blocking in cells exposed to enone and eneamide hit compounds. (**a**,**b**) MelJuSo-Ub^G76V^-YFP melanoma cells or KMS-11 multiple myeloma cells were exposed to the different hit compounds at 5 or 10 μM for 6 hours and extracts prepared for western blotting. The same filters were used to probe with different antibodies. BZ: bortezomib. Note the accumulation of K48-linked polyubiquitinated proteins in cells exposed to compounds; the lack of signal in two KMS-11 samples is due to strong cytotoxicity at 10 μM concentrations and loss of cells. (**c**) Glycerol gradient centrifugation of cellular lysates from exposed cells. Twelve fractions were collected from each gradient and subjected to western blot analysis. Note the presence of polyubiquitinated proteins in the proteasome fractions in the bottom (right) of the gradients (LLVY cleavage activity was found in the same fractions; not shown).

Hit compounds were tested on a number of different human tumor cell lines. Accumulation of K48-linked polyubiquitin proteasome substrates in melanoma, myeloma (KMS-11 and OPM-2) and HeLa cells (Fig. 2a,b and Supplementary Fig. 2a). Increased levels of K48-linked polyubiquitin chains were also observed using immunofluorescent staining of exposed HCT116 colon cancer cells (Supplementary Fig. 2b). Variable degrees of accumulation of p21^Cip1^/CDKN1A, a cyclin-dependent kinase inhibitor known to be degraded by the proteasome^39^, were observed in exposed cells (Fig. 2a,b and Supplementary Fig. 2a). We also observed increased levels of the inducible chaperone HSP70B’, consistent with the induction of proteotoxic stress, and expression of the Nrf-2 target HO-1 (heme oxygenase), suggesting induction of oxidative stress (Fig. 2a,b and Supplementary Fig. 2a). Glycerol gradient centrifugation experiments demonstrated that polyubiquitin chains accumulating in cells cosedimented with proteasomes (Fig. 2c), showing that polyubiquitinated proteins bind to ubiquitin receptors but are not processed further. The mechanism of proteasome inhibition therefore appears distinct from that of the dienone RA-190 (Supplementary Fig. 1a) which was described to bind the ubiquitin receptor Rpn13^40,41^. To examine whether proteasome subunit structure is affected, we used a cell line where PSMD14 (Rpn11) contains a tag that facilitates affinity purification^42^. Affinity purified proteasomes from exposed cells contained similar levels of USP14, UCHL5, PSMA2, PSMC2 and PSMD4 subunits as control cells (Supplementary Fig. 2c), showing that dissociation between 19S and 20S particles did not occur.

### Association between cell death and proteasome inhibition

**Figure 3:**
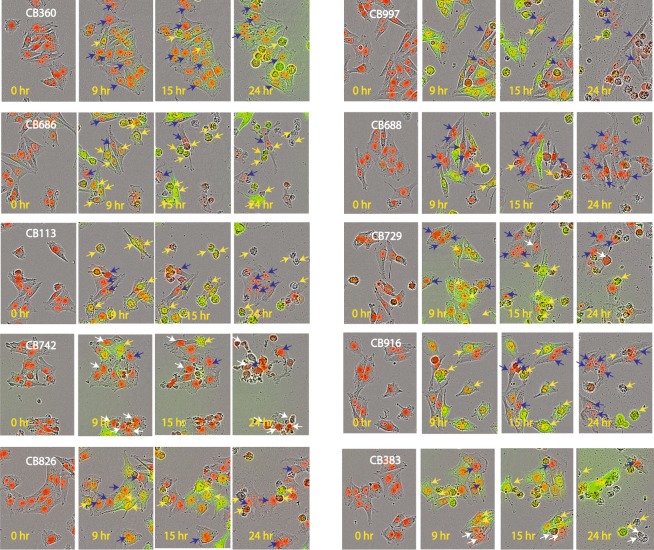
Association between proteasome blocking and cell death. MelJuSo-Ub^G76V^-YFP melanoma cells were exposed to the different hit compounds for 24 hours. An IncuCyte® instrument was used to monitor cells during exposure. Examples of cells becoming YFP positive and subsequently dying are shown with yellow arrows, examples of cells not becoming positive for the reporter and surviving treatment are shown with blue arrows, and examples of YFP-negative cells that died during incubation are indicated by white arrows. Note the variable degree of survival of cells that did not become positive for the Ub^G76V^-YFP reporter during drug exposure.

As described above (Fig. 1d), cells that became positive for the Ub-YFP reporter subsequently rounded up and lost their Ub-YFP protein content. If cell death is closely associated with proteasome inhibition one would predict that, at ~IC_50_ drug concentrations, cells that do not become Ub-YFP positive will survive. We analyzed time-lapse recordings of exposed MelJuSo-Ub^G76V^-YFP cells to examine this prediction. We indeed found that during exposure to some compounds, particularly **CB688**, **CB916** and **CB826**, cells that did not become Ub-YFP positive survived and proliferated (examples indicated by blue arrows in Fig. 3). However, other compounds, particularly **CB742** and **CB383**, displayed significant general non-proteasome-associated toxicity, i.e. also YFP-negative cells died during treatment (examples indicated by white arrows in Fig. 3). We conclude that proteasome inhibition is closely associated with subsequent cell death for some compounds, but not for others. A summary of the results from the analysis of time-lapse recordings are provided in Table 1.

**Table 1:** Summary of off-target activities and developmental toxicity.

Compound	Association between cell death and proteasome inhibition^&^	Developmental toxicity day 1–3	Developmental toxicity day 2–5
CB360	+++	−	−
CB997	++	−	+
CB686	+	+	+
CB688	++++	−	−
CB113	+++	−	−
CB729	+++	+	+
CB742	+	+	+
CB916	++++	−	−
CB826	++++	+	+
CB383	+	+	−

^&^General toxicity was evaluated by following the fate of the cells where proteasome inhibition was not observed. The number of cells/microscope field that remained negative for the Ub-YFP reporter was determined after 9 hours and the fate of these cells was determined (using time-lapse video recordings) after an additional 24 hours (cell death of Ub-YFP positive cells typically occurred at 12–14 hours, Fig. 1d). An increase in the number of Ub-YFP negative cells to >200% was scored as ++++, Ub-YFP(−) viable cell counts of 100–200% was scored as +++, Ub-YFP(−) viable cell counts of 50–100% scored as ++ and decrease to less than 50% of the number of Ub-YFP(−) observed at 9 hours scored as +. Cells did in no instance become Ub-YFP(+) at later times than 9 hours.

### Hit compounds inhibit proteasome deubiquitinase activity

**Figure 4:**
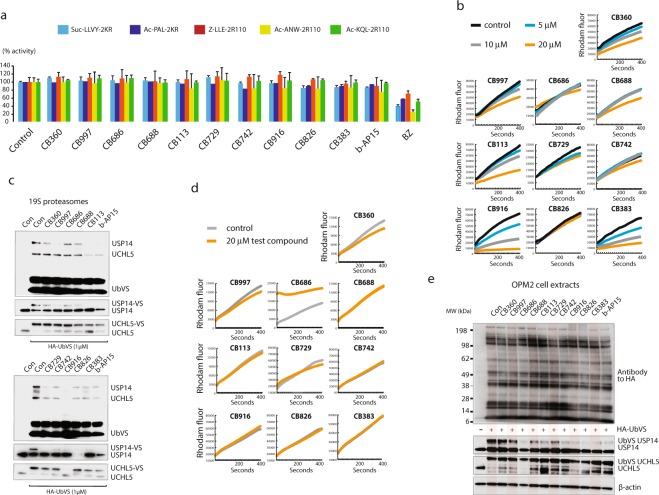
Inhibition of proteasome deubiquitinases. (**a**) Examination of 20S proteasome inhibition by hit compounds. Cell lysates (25 μg) were exposed to 20 μM of each compound except for bortezomib (BZ; 50 nM) for 5 min in assay buffer followed by the addition of substrates. The substrates Suc-LLVY, Z-LLE and Ac-KQL were used to assay β5c, β1c and β2c proteasome activity; the substrates Ac-PAL and Ac-ANW were used to assay β1i and β5i immunoproteasome activity. (**b**)19S proteasome preparations (10 nM) were incubated with ubiquitin rhodamine in the presence of the indicated compounds at 37 °C and fluorescence recorded; (**c**) 19S proteasome preparations (10 nM) were exposed to different compounds at 50 μM followed by labeling with HA-ubiquitin-vinylsulphone (HA-UbVS). The blots were probed with antibodies to HA, USP14 and UCHL5. Note the preferential loss of USP14 HA-UbVS labeling. The loss of recognition of USP14 following exposure to **CB826** was observed in repeated experiments and remains unexplained (this phenomenon was not observed using extracts (see below)). The previously described DUB inhibitor b-AP15^28^ was included as a reference. (**d**) Total cellular lysates from OPM-2 cells were incubated with ubiquitin rhodamine in the presence of 20 μM of the indicated compounds at 37 °C and fluorescence recorded. (**e**) OPM-2 cell extracts were incubated with compounds (20 μM) followed by HA-UbVS labeling. Filters were incubated with the indicated antibodies (also see Supplementary Fig. 3 where compounds were used at 50 μM).

None of the compounds were found to inhibit any of the proteolytic activities of the 20S proteasome (Fig. 4a). We next examined whether hit compounds inhibit 19S proteasome DUB activity using a general DUB substrate (Ub-Rhodamine). Significant inhibition of 19S DUB activity was observed for a number of hit compounds, particularly for **CB360**, **CB113**, **CB916** and **CB383** (Fig. 4b). In order to determine inhibition of individual DUB enzymes we used the DUB active site-directed probe ubiquitin vinyl sulphone (HA-UbVS)^43^ to label 19S proteasomes (Fig. 4c). Activity was either determined using antibodies to HA (top panel) or to USP14 or UCHL5 (lower panel). All hit compounds showed USP14 inhibitory activity in this assay, whereas inhibition of UCHL5 was generally less pronounced (Fig. 4c). Disparate results were obtained with compound **CB826**, i.e. no inhibition of Ub-Rhodamine cleavage activity but inhibition of Ub-VS labeling. The loss of detection of the USP14 protein on immunoblots (observed in repeated experiments using **CB826**) is not currently understood but could possibly be due to epitope masking. We conclude that all compounds studied here show inhibitory activity of USP14 with a subset also inhibiting UCHL5, without affecting 20S activity.

To examine whether hit compounds show general DUB inhibitory activity, we assayed cleavage of Ub-Rhodamine in total cell extracts. Inhibition of total cellular DUB activity was generally not observed at a concentration of 20 μM (Fig. 4d), a concentration where most compounds inhibited proteasome Ub-Rhodamine cleavage activity (Fig. 4b). Weak inhibition was observed using **CB360** and **CB729**. General inhibition of Ub-VS labeling of total cell extracts was not observed for any compound, including **CB360** and **CB729** (Fig. 4e). The same blots were used to probe with antibodies to specific DUBs. Inhibition of HA-UbVS labeling was observed for USP14, whereas labeling of USP5, USP7, USP48 and UCHL3 was not inhibited (Fig. 4e, Supplementary Fig. 3). Variable inhibition of UCHL5 was observed at 20 μM, inhibition being most pronounced for **CB113** (Fig. 4e). Compound **CB916** showed different UCHL5 inhibitory activity using cell lysates and 19S proteasomes (Fig. 4c,e**)** for reasons not currently understood. Dose response titrations using **CB729** is shown in Supplementary Fig. 3, showing inhibition of UCHL5 but not other DUBs tested. We conclude that the hit compounds do not show general DUB inhibitory activities. However, not all cellular DUBs were tested and some variability in labelling is observed in Fig. 4e. The finding of no general inhibition of Ub-Rhodamine cleavage using total cell extracts should also be interpreted with some caution since not all DUBs are expected to equally contribute to cleavage activity (or be expressed in HCT116 cells).

### Binding of hit compounds to USP14

We examined *in vitro* whether hit compounds bind to recombinant USP14 using isothermal titration calorimetry (ITC), intrinsic and extrinsic tryptophan fluorescence. Reducing conditions were used since USP14 could not be maintained in non-reducing conditions at the higher concentrations required for biophysical analysis. While limited solubility and large dilution enthalpies prohibited detailed quantitative ITC analysis for most hit compounds, significant direct binding was observed for compounds **CB826**, **CB916** and **CB997** (Supplementary Fig. 4a). Furthermore, saturable shifts in the intrinsic fluorescence of compounds **CB729** and **CB916** were observed in titrations with full-length USP14 and the USP14 catalytic domain, but not with the USP14 ubiquitin-like domain, indicating direct binding to the USP14 catalytic domain (Supplementary Fig. 4b).

**Figure 5:**
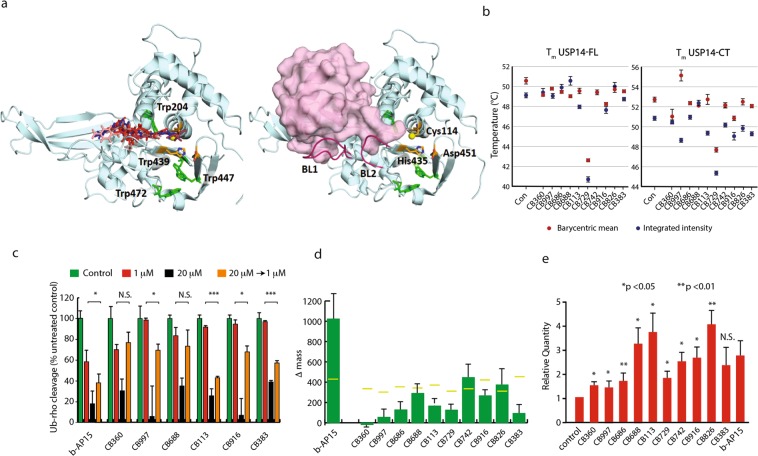
Binding of compounds to USP14. (**a**) *In silico* docking of different compounds to USP14 (left) and structure of the catalytic domain USP14 (PDB-id: 2AYO) in complex with ubiquitin aldehyde (right). Residues included in the catalytic triad are indicated in orange with the sulfur from Cys114 colored yellow, tryptophans in green and the blocking loops (BL1 and BL2) in purple. (**b**) Plot of T_m_ from thermal unfolding screen of full-length USP14 and its catalytic domain with 10 hit compounds analyzed with the barycentric mean and integrated intensity. (**c**) Dilution recovery of DUB activity. 19S proteasomes were exposed to DMSO, 1 μM or 20 μM for 20 min at 37 °C and followed by analysis of Ub-rhodamine cleavage activity. Samples were diluted 20-fold where indicated (20 μM - > 1 μM); *p < 0.05, ***p < 0.005. (**d**) MALDI-TOF analysis of incubation of USP14 with hit compounds. Recombinant USP14 (20 μg) was incubated with 20 μM compound for 1 hour at 37 °C and analysed by MALDI-TOF. Shifts in molecular masses were calculated (n = 3, mean + S.E.M.). Horisontal bars show the shifts in mass expected from binding of one molecule. (**e**) thermostability of UPS14 analysed by CETSA (cellular thermal shift assay)^46^. Statistic significant was calculated by matched pairs t-test (n = 6).

To enable the evaluation of binding effects of all compounds under reducing conditions, we used USP14 intrinsic fluorescence. The USP14 catalytic domain contains four tryptophan residues, where three (Trp439, Trp447, Trp472) are close to the protein surface and in the vicinity of the catalytic triad, and Trp204 is buried in the domain core (Fig. 5a). Effects on the USP14 thermal melting point (T_m_) were evident in the full-length protein, but more pronounced in the USP14 catalytic domain (Fig. 5b). All compounds significantly affected the thermal stability of either or both of USP14-CT and USP14-FL. The integrated fluorescence peak intensity, which is primarily affected by surface quenching effects, was more sensitive to the presence of hit compounds than the peak barycentre, which is a better readout of protein folding. The hit compound **CB729** stands out as pronouncedly destabilizing for both the catalytic domain and the intact protein. To evaluate possible binding sites for hit compounds to USP14, *in silico* docking of hit compounds to the catalytic domain of USP14 was performed using ICM-Pro provided by Molsoft (www.molsoft.com). The ICM-pocket finder was used to identify pockets with drug-like (DLID) scores > 0.5^44^ in 2AYO. The tentative binding pocket in 2AYO is located close to the active site and houses the C-terminal tail of ubiquitin in the Ub-loaded state (Fig. 5a). All compounds could be successfully docked to the tentative binding pocket, with best docking scores ranging from −39.3 (**CB383**) up to −19.1 (Supplementary Table 1). The docking of the five compounds with the lowest docking scores is illustrated in Fig. 5a. Hit compound binding to the identified pocket would likely counter protein stabilization by BL1 anchoring into the same groove and is thus in agreement with destabilization of the USP14 protein as observed by fluorescence (Fig. 5b). Similarly, such hit compound binding to this grove would agree with the observed inhibition of HA-UbVS labeling (Fig. 4c) as well as inhibited enzyme activity using a Ub-Rhodamine substrate (Fig. 4b), since both HA-UbVS and Ub-Rhodamine bind to the same pocket. These findings are consistent with recently published data for binding of the inhibitor IU1 to the thumb-palm cleft region of the USP14 catalytic domain^45^.

### Reversible and irreversible binding to USP14 under cellular, non-reducing conditions

Dilution recovery experiments were performed in order to examine whether hit compounds show reversible binding to USP14. Compounds were incubated with 19S proteasomes at 37 °C followed by dilution. USP14 activity could be recovered after 20 minutes of incubation (longer periods were not used) for two (**CB360** and **CB688**) out of six compounds that could be tested in this assay but was incompletely recovered for four compounds and for b-AP15 (Fig. 5c). In order to examine whether screening hits were capable of covalent binding to USP14 we incubated recombinant USP14 with hit compounds and then assayed USP14 molecular mass by MALDI-TOF under non-reducing conditions (Fig. 5d). The extent of molecular mass shifts was dependent on the length of incubation and generally resulted in smaller molecular mass shifts than expected. Results can therefore only be interpreted in terms on whether compounds bind to USP14 or not. Increases in the molecular mass of USP14 were observed for all compounds except **CB360** that did not induce any detectable shift. Together with the dilution-recovery assay, we conclude that hit compounds show evidence of irreversible enzyme inhibition and covalent reactivity to USP14, as expected from their chemical reactivity. There is no clear correlation between lack of reactivation of 19S proteasome DUB activity and covalent attachment of hit compound to recombinant USP14, possibly due to the use of different sources of enzyme and experimental conditions.

To analyze effects on USP14 under conditions where DUBs are bound to proteasomes in cells, we used a cellular thermal shift assay (CETSA)^46^ where the presence of USP14 in the soluble fraction of living cells is analyzed as a function of temperature. USP14 was increasingly observed in the soluble fraction in the presence of 9/10 compounds (Fig. 5e). In CETSA, such results are usually interpreted as thermostabilization of the protein, but since the proteasome is known to unfold at 53 °C^47^ and USP14 does not aggregate on unfolding *in vitro* as judged by light scattering (unpublished observations), the apparent thermal stabilization may also be due to dissociation of USP14 from the proteasome occurring at 53 °C (but apparently not at lower temperatures (Supplementary Fig. 2c). There was no obvious relationship between stronger USP14 thermostabilization (observed for **CB688**, **CB113** and **CB826**) and evidence of irreversible enzyme inhibition (Fig. 5c). Given the uncertainty of the biophysical interpretation of the experiment, we still conclude that the majority of the selected compounds affect USP14 or the proteasome in cells exposed to 5 μM compound.

### Inhibition of TrxR and determination of compound electrophilicity

**Figure 6:**
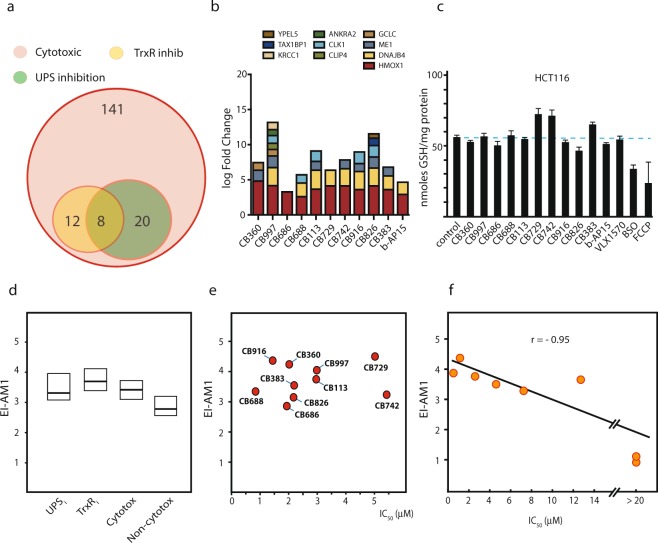
Analysis of oxidative stress, electrophilicity and off-target activity. (**a**) Inhibition of thioredoxin reductase (TrxR) by cytotoxic compounds; 20/141 cytotoxic compounds were found to inhibit TrxR (Supplementary Fig. 5**)**. (**b**) Induction of the expression of Nrf-2 target genes^74^. Data obtained by microarray analysis (see Fig. 7). (**c**) Effects of hit compounds on cellular glutathione levels following exposure to compounds for 6 hours. HCT116 cells were exposed to 5 μM of each compound for 6 hours and glutathione measured. (**d**) Electrophilicity indices of various classes of compounds; shown are medians and quartiles. UPS inhibitors (the 28 compounds identified here), TrxR inhibitors, cytotoxic and non-cytotoxic compounds (**a** randomly selected set of 20). The electrophilicity indices of the β-carbonyls of the different compounds were calculated utilizing the MOE software as described in Methods. (**e**) Relationship between electrophilicity indices ω and IC_50_ of the compounds identified in the study. IC_50_ values were determined using HCT116 cells by MTT assay (72 hours exposure). (**f**) Relationship between electrophilicity indices and IC_50_ values of compounds in the VLX1500 series. These are compounds with the general structure of **VLX1570 (**Supplementary Fig. 1a**)**^51^. The electrophilicity of this series differs due to the presence/absence of electron-drawing groups on the aryls.

We performed a counter screen for inhibition of thioredoxin reductase (TrxR), an important selenoprotein in antioxidant defense and control of cellular redox regulation. This enzyme contains redox active cysteine residues within an N-terminal domain as well as a highly nucleophilic selenocysteine residue at the C-terminus that is known to be easily targeted by electrophiles^48,49^ and its inhibition typically leads to activation of Nrf-2^50^. We found that 20/141 (14%) of compounds inhibited TrxR and that eight of the 28 UPS inhibitors also inhibited TrxR (Fig. 6a, Supplementary Fig. 5**)**. None of these eight compounds belonged to the set of the 10 most potent UPS inhibitors. We observed increased expression of genes known to the targets of the Nrf-2 transcription factor by most of the hit compounds (Fig. 6b). These finding demonstrate a higher selectivity in targeting of thiol-dependent enzymes by these electrophilic compounds, some of which were also classified as PAINS, than envisioned.

The observation of increased expression of Nrf-2 target genes pointed to induction of oxidative stress. We examined the levels of reduced glutathione (GSH) in cells exposed to IC_90_ doses of the different hit compounds for six hours. The results did not show any pronounced effects on GSH levels in the exposed cells (Fig. 6c). Compound **CB826** reduced GSH content whereas compounds **CB729** and **CB742** induced increases in intracellular GSH (Fig. 6c).

A common method used in predictive toxicology to describe the reactivity of an electrophilic compound is the molecular electrophilicity index (ω). The electrophilicity index is a descriptor of reactivity that allows a quantitative classification of the global electrophilic nature of a molecule within a relative scale. The electrophilicity indices ω of the β–carbonyls of the different compounds were calculated utilizing the MOE software (Fig. 6d,e). The median electrophilicity index ω_M_ of the 28 UPS inhibitors was 3.3 eV, similar to that of TrxR inhibitors (ω_M_: 3.6 eV) and generally cytotoxic compounds (ω_M_: 3.4 eV) but slightly higher compared to the EIs of non-toxic compounds (ω_M_: 2.8) (Fig. 6d). There were, however, non-toxic compounds with ω of >3.6 eV. Importantly, there was no apparent correlation between ω and antiproliferative activities of the hit compounds (Fig. 6e). We also determined the electrophilicity indices of compounds in the dienone-containing and structurally related VLX1500 series^51^. These compounds have the same basic structures but variable ω due to the presence/absence of electron drawing groups on the phenyl groups. In this series there was a strong correlation between ω and biological activity (Fig. 6f).

### Induction of ER stress and apoptosis

**Figure 7:**
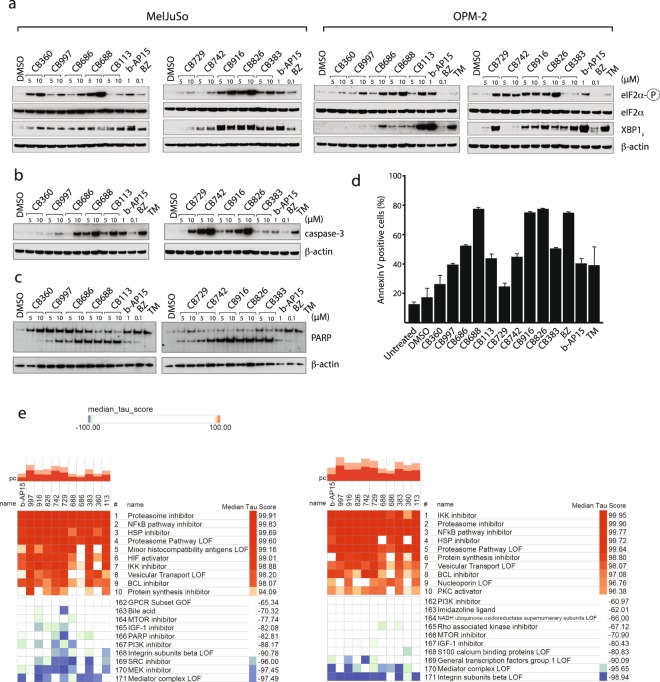
Analysis of the phenotypic response to hit compounds. (**a**) MelJuSo or OPM-2 cells were exposed to compounds as indicated for 6 hours and extracts were processed for immunoblotting. Note the induction of eIF2α phosphorylation and the protein products translated from the spliced form of XBP1 by all compounds in MelJuSo cells and by most compounds in OPM-2 cells. TM = tunicamycin. (**b**) Western blot analysis of active caspase-3 and (**c**) PARP cleavage following exposure of OPM-2 cells to test compounds for 6 hours. (**d**) Analysis of annexin V binding to exposed OPM-2 cells. Cells were exposed to the compounds at 5 μM for 18 hours, incubated with fluorescent annexin V and analyzed by flow cytometry (shown is total annexin V signal). (**e**,**f**) Cmap analysis of responses to treatment. MCF7 cells were exposed to the different compounds for 6 hours and gene expression analysed using Affymetrix microarrays. Gene signatures were uploaded into the Cmap data base^53^. Compounds are compared to pertubational classes and ranked for similarity by Kendall’s tau coefficient. Higher median tau score indicates a high similarity between the compound and the pertubational class of compounds. (**e**) Ranked similarity in gene expression patterns of MCF7 cells treated with hit compounds compared to gene expression patterns of all cell types in the CMap database, analyzed on the level of pertubational classes. (**f**) Ranked similarity in gene expression patterns of MCF7 cells treated with hit compounds compared to gene expression patterns of MCF7 cells in the CMap database, analyzed on the level of pertubational classes.

Proteasome inhibitors commonly induce ER stress^52^ and we examined whether hit compounds induced phosphorylation of eIF2α and the spliced variant of XBP1. We indeed found that all ten compounds (Fig. 7a) induced both markers in MelJuSo cells, although the extent of induction varied. This variation was even more pronounced in OPM-2 myeloma cells (Fig. 7a). ER stress is associated with induction of apoptotic cell death and we found cleavage of pro-caspase-3, cleavage of PARP and annexin V positivity in OPM-2 cells exposed to the hit compounds (Fig. 7b–d), consistent with induction of apoptosis.

### Analysis of gene expression changes shows signatures characteristic of UPS inhibitors

The cellular response to treatment will give a read-out of the major mechanism of compound action^53,54^. We examined the gene expression signature of cells exposed to hit compounds and to the enone DUB inhibitor b-AP15^28^ (Fig. 7e,f and Supplementary Fig. 6a,b). The CMap resource was used for this analysis^53^ is mainly based on the MCF-7 cell line, which was therefore used for this experiment. The analysis showed that IKK inhibition, proteasome inhibition and NF-κB inhibition resulted in the highest scores for the compounds and that the profiles were very similar to that of b-AP15. Differences were observed, consistent with expected differences in off-target activities, but these differences did not reflect the observed non-proteasome inhibitor-associated cell death described above (Table 1). We also performed gene ontology (GO) enrichment analyses and gene set enrichment analyses (GSEA) for the different compounds^55^. The “protein refolding” GSEA profiles all showed a similar degree of enrichment for all compounds (Supplementary Fig. 6b). We conclude from these results that the major cellular response to this set of chemically diverse compounds appears to be related to effects on protein homeostasis.

### *In vivo* anti-neoplastic activity

**Figure 8:**
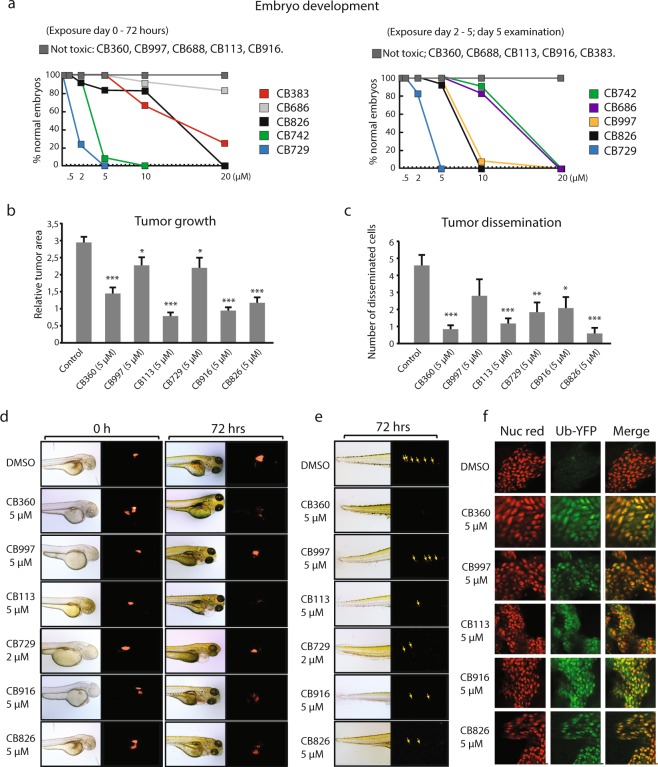
Antineoplastic activity in the zebrafish embryo model. (**a**) Effects on zebrafish embryo development. Compounds were added to the water and the number of normal embryos determined after incubation; (left) incubation day 0 to day 3 (72 hours); (right) incubation day 2 to day 5 (72 hours). (**b**,**d**) Assessment of tumor cell growth in zebrafish embryos. Embryos (n = 20) were injected with labeled MelJuSo cells and fluorescence determined after injection (basal level) and after 72 hours (average + S.E.M.). Not all compounds were tested due to observations of precipitation. (**c**,**e**) Assessment of tumor cell dissemination in zebrafish embryos. Embryos (n = 20) were injected with labeled MelJuSo cells and labeled cells in dorsal regions were recorded after 72 hours (average + S.E.M.). (**f**) MelJuSo-Ub^G76V^YFP cells were injected into zebrafish embryos and allowed to establish for 72 hours. The indicated compounds were added to the water and yellow and red fluorescence recorded after 12 hours. Note the induction of Ub^G76V^YFP by each compound. Statistical significance calculated by t-test; *p < 0.05, **p < 0.01, ***p < 0.001.

In order to examine toxicity and potential antineoplastic activity we used the zebrafish embryo model^56^. Zebrafish has been advocated as a suitable model for toxicity assessment of anticancer compounds^57^. Interestingly, four compounds did not affect embryonal development when added at 20 μM during the first three days of development or during day 2–5 (**CB360**, **CB688**, **CB113** and **CB916**). Three of these compounds were classified as PAINS. The remaining compounds showed variable developmental toxicity, **CB729** being most potent (Fig. 8a). To examine effects on tumor cells, embryos were injected with dye-labeled MelJuSo cells after eight hours of development, compounds were then added and effects evaluated after an additional 72 hours. We tested three compounds that did not induce embryonal toxicity at any concentration (**CB360**, **CB113** and **CB916**) and three other compounds (**CB997**, **CB729**, and **CB826**). Compounds were used at 5 μM, except for **CB729** that was used at 2 μM. Tumor cell growth was significantly inhibited by all six tested compounds, most effectively by **CB113**, **CB916** and **CB826** (Fig. 8b,d). The ability of injected tumor cells to spread to distant sites (a model for metastatic dissemination^58^) was also examined (Fig. 8c,e). All compounds except **CB997** significantly inhibited tumor cell dissemination. In order to determine whether the effects on growth and dissemination were associated with proteasome inhibition *in vivo* we examined stabilization of the Ub^G76V^-YFP reporter protein (Fig. 8f). Tumor cells were injected into zebrafish embryos and compounds were then added after 72 hours. Embryos were examined by confocal microscopy after an additional 12 hours. All five compounds examined were found to induce Ub^G76V^-YFP reporter expression (Fig. 8f). We conclude from these experiments that all five tested compounds were able to penetrate zebrafish embryos and to inhibit proteasome function in the injected melanoma cells.

## Discussion

We here report that in a set of 141 cytotoxic α,β-unsaturated compounds identified by screening, one fifth induced the accumulation of polyubiquitinated proteasome substrates. The cellular response to the ten compounds studied in detail was characterized by proteotoxic stress, ER stress and induction of apoptosis. Gene expression analysis revealed that the cellular response to all compounds was characteristic of proteasome inhibitors, protein folding being the most enriched gene ontology (Fig. 7e,f, Supplementary Fig. 6a). The protein folding GSEA enrichment scores were overall similar to that observed for the b-AP15 compound (Supplementary Fig. 6b). Analysis of inhibition of processing of a proteasome reporter substrate and induction of cell death using time-lapse microscopy showed strong associations for a number of compounds. Compounds **CB688**, **CB916** and **CB826** showed the strongest association between cytotoxicity and inhibition of proteasome substrate processing. Two of these compounds (**CB688** and **CB916**) did not induce developmental toxicity and showed antineoplastic activity in the zebrafish model. **CB360** and **CB113** also showed strong associations between cytotoxicity and proteasome inhibition and did not induce developmental toxicity. None of the compounds identified showed potencies <1 μM in cellular assays and these hits would require lead optimization to be used as cancer therapeutics. To summarize, the result show that compounds containing Michael acceptor groups can induce a specific pharmacological response, that of proteasome inhibition. This response is closely associated with cell death, most likely due to the elevated sensitivity of tumor cells to UPS inhibition.

The b-AP15/RA-9/G5 analogue RA-190 (Supplementary Fig. 1a) described to bind cysteine 88 of ubiquitin receptor Rpn13 in the 19S regulatory particle leading to inhibition of proteasome function^40,41^. We here found that polyubiquitinated proteins accumulate on proteasomes in cells exposed to the hit compounds, not consistent with blocking of polyubiquitin binding to ubiquitin receptors but not excluding other effects on interactions of ubiquitin receptors with polyubiquitinated substrates. We were able to document inhibition of the cysteine DUB USP14 by all hit compounds. Inhibition or knock-down of USP14 has been reported to lead to loss of cell viability of some tumor cells^59,60^, but not in others^61^ and not in normal cells^62,63^. The USP14 inhibitor IU1 in fact increases the viability of cells under conditions of oxidative stress^62^. The strong cytotoxicity observed here is unlikely to be a result of inhibition of USP14 alone, and other targets in the UPS, such as UCHL5, are likely to contribute to the response. Some of the compounds were found to inhibit UCHL5 and it is known that knockdown of both USP14 and UCHL5 severely inhibits proteasome processing of polyubiquitinated proteins^61,64^. We did not observe inhibition of total cellular DUB activity or different DUBs examined such as USP7. It was recently reported that different USP14 inhibitors of the IU1-series bind to the same pocket identified here^45^. These molecules bind to a site that is distant from the catalytic center, blocking access of the C- terminus of ubiquitin. Furthermore, recently identified high-affinity USP7 inhibitors^65^ bind to the same thumb-palm cleft that was targeted in our docking experiments (Fig. 5a). The targeted USP7 region is highly similar to USP14 in the floor of the binding cleft, where three conserved aromatic residues provide hydrophobicity. In the roof of the cleft, which holds the so-called “switching loop”, specific side chains form hydrogen bonds to the conserved PyrzPPip moiety of the USP7 small-molecule inhibitor^65^. Notably, the “switching loop” region is not conserved in sequence or structure between USP7 and USP14 (Supplementary Fig. 4c), and in agreement, the recent highly specific USP7 inhibitory molecules do not affect USP14 at all^65^. The synthetic compounds identified here show similar properties as a number of widely studied α,β-unsaturated carbonyl-containing natural products such as curcumin, withaferin A, celastrol, piperlongumine and gambogic acid (Supplementary Fig. 1a)^14–20^. Curcumin, a constituent of turmeric, is possibly the most studied natural product not registered as a drug^66^ and there are >4,700 published studies on PubMed in the field of cancer research. A plethora of biological activities have been reported to be associated with curcumin^11^, many of which probably due to the multiple mechanisms for assay interference offered by its structure^4,66^ but may also, at least partly, be explained by inhibition of proteasome function^20,30^ and also by TrxR1 inhibition^67^. Proteasome inhibition will lead to a multitude of effects on cellular signaling systems and homeostasis and TrxR1 inhibition will lead to either cytotoxic effects or Nrf2 activation in normal cells. We note that NFκB inhibition, a well-described down-stream effect of proteasome inhibition, has been reported in >800 PubMed articles on curcumin. Our result that UPS inhibition is not a unique property of natural products containing Michael acceptors but appears to be a relatively common property of this class of small molecules, including synthetic compounds.

Counter-screening is of pivotal importance in screening projects for exclusion of compounds that form colloids and/or are pan-inhibitory^7,68^ and was of particular importance in this project. We extended our analyses to include thioredoxin reductase, known to be especially prone to electrophilic attack by α,β-unsaturated carbonyls due to its nucleophilic active site Sec residue^67^. We were surprised to find that only 20/141 cytotoxic compounds inhibited TrxR. Our results showed that UPS and TrxR inhibition by such compounds not necessarily correlate with each other, but that inhibition of either system can be found with 34% of cytotoxic compounds of this class. We conclude that our screen identified molecules that inhibit proteasome substrate processing and for which a close association between cytotoxicity and proteasome inhibition could be demonstrated. This observation sheds light on the reports in the literature of natural products eliciting this type of response. Whereas the expected reactivity of these types of compounds makes them unsuitable as biological probes, their ability to induce a pharmacological response characteristic of proteasome inhibitors may potentially be used for cancer therapy.

## Materials and Methods

### Materials

The compound library was purchased from ChemBridge. Selection of enones was performed by Reg Richardson at ChemBridge with a LogP restriction of between 1 and 4. All compounds were dissolved in dimethylsulphoxide (DMSO) and used at a final concentration of 0.5% DMSO; control wells received solvent only (final concentration 0.5% DMSO).

### Cell culture and screening

A panel of different human tumor cells were used in the study. Myeloma cells are sensitive to proteasome inhibitors and were particularly important to use in the present study. KMS11 and OPM-2 myeloma cells were maintained in RPMI1640 medium supplemented with 10% fetal calf serum. The proteasome reporter cell line MelJuSo Ub^G76V^-YFP^69^ and HeLa cells were cultured in Dulbecco’s modified Eagle’s medium/10% fetal bovine serum. The human colon carcinoma cell line HCT116 has been previously used in studies of proteasome DUB inhibitors^28^ and was maintained in McCoy’s 5A medium. The breast cancer cell line MCF-7, used for the genomics-based mechanistic analysis^53^, was cultured in Minimum Essential Medium Eagle with 1 mM sodium pyruvate and supplemented with 10% fetal calf serum. Cells were maintained at 37 °C in 5% CO_2_. Screening was performed using MelJuSo Ub^G76V^-YFP cells transfected with a Nuclear Red marker (Essen BioScience Inc., Ann Arbor, MI). Cells were plated in black optically clear bottom 96-well ViewPlates (Sarstedt, Nürnbrecht, Germany) over-night and then treated with 5 μM compound. Cell numbers are reflected in the strength of red signal. Compounds that block the UPS induce accumulation of YFP in these cells, and the generated fluorescence was continuously detected in an IncuCyte FLR instrument (Essen BioScience) that captures images of the cells over time.

### Western blotting

Cell extract proteins were resolved by Tris-Acetate 4–12% SDS-PAGE gels (Invitrogen, Carlsbad, CA) and transferred onto nitrocellulose membranes, which were incubated overnight to antibodies, washed and incubated with HRP-conjugated anti-rabbit Ig (7074S) or anti-mouse Ig (7076p2) (Cell Signaling Technology, Danvers, Mass) for 1 h. Source of antibodies: K48-linked polyubiquitin (05-1307)(Apu2, Millipore), Hsp70B′ (HPA028549, Sigma-Aldrich), heme oxygenase-1 (610713, BD Biosciences), GFP (2955S), p21^Cip1^ (3946S)(Cell Signaling Technology), β-actin (A5316, Sigma-Aldrich; 1:10,000), PSMD4 (#3336S), PSMA5 (#2458), PSMC2 (#14395S), PSMD14 (#4197S), PSMA2 (#2455S), all from Cell Signaling, USP14 (A300-920A), UCHL5 (A304-099A), USP5, (A301-542A), USP48 (A301-190A), USP7 (A300-033A), UCHL3 (A302-948A, all from Bethyl Laboratories, HA-TAq (3724S, Cell Signaling), active caspase-3 (99661S, Cell Signaling) and anti-PARP (556362) from Bethyl Laboratories), phospho-eIF2α(9721S), eIF2α (9722S) and XBP1S (12782S) (ER stress sampler kit, Cell Signaling Technologies).

### Glycerol density gradient fractionation

Glycerol gradient fractionation was conducted on 15-35% linear glycerol gradients containing 25 mM Tris-HCl pH 7.6, 5 mM MgCl_2_, 1 mM dithiothreitol and 2 mM ATP. Cell lysates containing 500 μg of protein were loaded on glycerol gradients and samples were centrifuged at 26000 rpm for 28 hours at 4 °C in an Optima L-80 XP Ultracentrifuge using a SW41 rotor (Beckman Coulter). Fractions were collected (24 × 500 μl) and proteins were precipitated in cold acetone for 1 h at −80 °C. Paired fractions were pooled and the 12 samples were prepared and used for immunoblotting.

### Measurement of proteasome activity and deubiquitinase activity

*In vitro* proteasome activity assays were performed using total cellular extracts of OPM-2 cells (25 μg) in reaction buffer (25 mM Hepes, 0.5 mM EDTA, 0.03% SDS). We used the substrates Suc-LLVY-2R110 (β5c), Z-LLE-2R110 (β1c) and (Ac-KQL)2R110 (β2c) from AAT Bioquest (Sunnyvale, CA). A Tecan Infinite 200 reader equipped with 498 nm excitation and 520 nm emission filters was used for detection. For DUB inhibition assays, Ubiquitin-Rhodamine 110 (Rh110) (Boston Biochem, Cambridge, MA) was used as a substrate and either total cell lysates or 19S proteasomes (Boston Biochem) as source of enzyme using procedures described previously^28^. Labeling with ubiquitin-vinylsulphone was performed as described^64^ using reagent from Boston Biochem.

### Modeling method

*In silico* docking of hit compounds to the catalytic domain of USP14 were performed using ICM-Pro provided by Molsoft. A DLID score > 0.5 is considered druggable (http://www.molsoft.com/gui/3d-predict.html2. http://www.molsoft.com/gui/start-dock.html). Each hit compound was removed. ICM pocket finder were used to predict pockets and the pocket with highest DLID (drug-like density) score were then chosen fund were docked with a thoroughness of 10. From the output, a score of the docking is obtained which is a function of several parameters. A score of −32 or lower is considered good. *In silico* docking were performed between USP14 in two conformations, one with two loops (BL1 and BL2) in a closed (PDB-id: 2AYN) and one open (PDB-id: 2AYO) conformation. The ICM-pocket finder could not recognize any pocket with sufficient DLID-score in the closed conformation, therefor the open conformation was used for the docking. The best DLID-score were obtained from pocket 1 (Supplementary Table 1) to which all hit compounds were docked. The docking score ranged from −39.30 to −19.13 with only compound **CB383** obtaining a score below −32 (Supplementary Table 1). The structure and docking is illustrated in Fig. 5a where it binds to the same site as the C-terminal tail of ubiquitin aldehyde.

### Protein production

His-tagged USP14 and its two domains USP14-CD (residue 91–494) and USP14-Ubl (residue 1–80) were separately cloned into pNIC-28-Bsa4 expressed in *E. coli* BL21(DE3) Ros-2 cells. Cells were grown in LB-medium overnight at 37 °C, transferred to TB-medium and incubated at 37 °C to O.D. 0.8. The culture was induced with 0.5 mM IPTG overnight at 18 °C. The cell pellet was resuspended in lysis buffer (20 mM HEPES, 500 mM NaCl, 10 mM imidazole, 5% glycerol, 0.5 mM TCEP, 5 units recombinant DNAse I and 1 EDTA-free protease inhibitor cocktail tablet per 75 mL) and lysed using sonication. The soluble material was loaded onto Ni-bead column equilibrated with wash I buffer (20 mM HEPES, 500 mM NaCl, 10 mM imidazole, 5% glycerol, 0.5 mM TCEP), washed with wash I and wash II buffer (20 mM HEPES, 500 mM NaCl, 20 mM imidazole, 5% glycerol, 0.5 mM TCEP) and eluted with elution buffer (20 mM HEPES, 500 mM NaCl, 250 mM imidazole, 5% glycerol, 0.5 mM TCEP). Eluted protein was cleaved and dialyzed overnight with TEV protease in dialysis buffer (20 mM HEPES, 300 mM NaCl, 5% glycerol, 0.5 mM TCEP) at 4 °C. The cleaved protein was loaded onto Ni-bead column equilibrated as previously described. The flow through was collected and loaded onto a Hiload 16/600 Superdex 75 pg column (GE Healthcare) equilibrated with dialysis buffer. The protein was aliquoted in fractions of 10–70 μM, flash-frozen in liquid nitrogen and stored at −80 °C.

### Isothermal titration calorimetry (ITC)

The ITC measurements were performed using Microcal PEAQ-ITC (Malvern) at 298 K with USP14 at concentration of 10–25 μM in 20 mM HEPES pH 7.5, 300 mM NaCl, 10% Glycerol, 5% DMSO and 2 mM TCEP. 13–20 injections of 2–3 µl of hit compounds (100–500 µM in 100% DMSO) to a cell volume of total 300 μl. The raw data were collected and analyzed using the Malvern MicroCal Software for PEAQ-ITC Analysis.

### Fluorescence spectral scan

Spectral scans of full-length USP14 and its two domains with hit compounds were carried out with the CLARIOstar multimode microplate reader by scanning excitation and emission wavelength for ligand alone and with full length protein. Hit compounds CB729 and **CB918** yielded the most significant change of intensity and were thus chosen for further titrations. Compound **CB918** was excited at 450 nm and emission was recorded between 570–700 nm and **CB729** was excited at 375 nm and emission were recorded between 500–600 nm. Samples were prepared with protein and hit compound concentration at 5 μM in 20 mM HEPES, 100 mM NaCl, 0.5 mM TCEP, 5% glycerol and 5% DMSO. 100 μl was transferred to a 96 well microplate for measurements.

### Thermal unfolding screen

A fluorescence-based thermal unfolding screen was conducted in the temperature range 20–90 °C using an Optim 1000 fluorimeter (Avacta Analytical Ltd). For each sample, 6 µM full-length or C-terminal domain USP14 was analyzed in tenfold molar excess of the tentative small-molecule binder. Integrated intensity and barycentric mean in the emission range 280–460 nm were calculated from fluorescence emission after excitation at 280 nm. Tm was calculated with the software CDpal^70^.

### MALDI-TOF mass spectrometry

Recombinant USP14 protein was desalted to remove interfering reducing agent on 30 kDa cut-off spin columns (Merck) and resuspended in water. For MALDI-TOF analysis of compound binding, 10 µg USP14 protein was incubated with 10 µM of selected compounds for 45 minutes at room temperature. Samples were then desalted to remove unbound compound on 30 kDa cut-off spin columns and resuspended in water. Approximately 0.25 µg sample was loaded onto polished stainless steel MALDI-TOF plates with sinapic acid (3,5-dimethoxy-4-hydroxycinnamic acid, Sigma) as matrix substance and air-dried until completely dry. Whole protein mass spectra were acquired on an UltrafleXtreme MALDI-TOF mass spectrometer (Bruker Daltonics) instrument operated in the linear positive ion mode with flexControl software (Version 3.4, Bruker Daltonics). Each spectrum was the accumulation of approx. 5000 laser shots for the sample spots. The MS spectra were externally calibrated using the Protein Calibration Standard II mixture (Bruker Daltonics). The MS spectra obtained were analyzed using flexAnalysis software (Version 3.4, Bruker Daltonik).

### CEllular thermal shift assay (CETSA)

CETSA was performed as described^46^. Each sample was suspended in PBS supplemented with protease inhibitors after they had been washed in PBS. Samples were at 53 °C for 3 min, incubated at room temperature for 3 min, and then snap-frozen in liquid nitrogen. Samples were freeze-thawed twice and centrifuged at 20,000 g for 20 min at +4 °C and supernatants analyzed by immunoblotting.

### Glutathione determination

Cells were washed in PBS, detached by trypsin and washed with PBS. Cells were then lysed and assayed in accordance with the protocol for Fluorimetric Glutathione Assay kit (CS1020, Sigma). GSH levels in the cells were determined by use of a GSH standard curve, and all samples were measured in triplicate. The readout was performed using Tecan Spark 10 microplate reader, and the data was analysed using GraphPad Prism 7.

### Calculation of electrophilicity indices

Electrophilicity indices were determined by applying the equation (ω = µ^2^/2η = E_HOMO_^2^ + 2 E_HOMO_ E_LUMO_ + E_LUMO_^2^/4 (E_LUMO_ − E_HOMO_)) where µ is the molecular chemical potential and η the molecular hardness, both of which can be replaced by the energies of the highest occupied molecular orbital (E_HOMO_) and the lowest unoccupied molecular orbital (E_LUMO_), via application of Koopmans’ theorem^71^. The HOMO and LUMO energy descriptors were calculated utilizing the semi-empirical Austin model 1 (AM1) level of theory with the MOPAC 7.0 engine (Molecular Operating Environment (MOE), 2013.08; Chemical Computing Group ULC).

### Microarray analysis

In order to compare response in RNA expression in our hit compounds compared to other pharmaceutical compounds, CMap and Gene ontology analysis was envisioned. To ensure optimal comparability, MCF-7 cells were used and guidelines from the original protocol were followed^53^. MCF-7 cells were treated for 6 hours with hit compounds at 2x [IC50] as determined by MTT assay. Cells were then lysed, and RNA was purified, using the RNeasy Plus Mini Kit following manufacturer’s instructions (Qiagen, Hilden, Germany). 500 nM of purified total RNA was further processed for microarray analysis using the GeneChip WT PLUS Reagent Kit according to manufacturer’s instructions (Affymetrix, Santa Clara, CA, USA). Prepared RNA was hybridized to GeneChip HuGene 2.0 ST chips for 16 h using the GeneChip Hybridization Oven 645, washed and stained using the GeneChip Fluidics Station 450 using the FS450_0002 protocol and scanned with a GeneChip Scanner 3000 7G (both Affymetrix). Data analysis was performed using R 3.3.2 and RStudio 1.0.143, using packages pd.hugene.2.0.st, oligo, limma, rColorBrewer. Pheatmap, ggplot2 and affycoretools (all retrieved from Bioconductor.org). Unannotated probes were omitted from further analysis. Only sample sets that passed quality control were used for further analysis. For CMap analysis the top 100 up or downregulated valid genes were uploaded into the online cMAP analysis query tool and analysed using the L1000 platform^54^ hosted on clue.io. Gene expression patterns were compared to pertubational classes present in the analysis database and heatmaps were produced using the ICV tool hosted on clue.io. For GSEA analysis, expression data of all probes were analyzed and visualized using the GSEA v2.0 tool. Genes were ranked according to signal-to-noise calculation where three repeats per condition were available, otherwise ratio-of-classes was used.

### Annexin V labeling

Quantification of apoptosis by Annexin-V and propidium iodide staining and flow cytometry was performed. Cells were cultured at density of 5 × 10^5^ cells/mL, then exposed to compounds as described using DMSO as control. Cells were harvested and washed in PBS twice, and stained with fluorescein-conjugated annexin-V and PI (BD-Biosciences, San Jose, CA).

### Developmental toxicity assay

At 4 hours post fertilization (hpf), alternatively 48 hpf as indicated in the text, 20 zebrafish embryos were incubated in 60 mm in diameter tissue culture dishes with 15 mL of E3 embryo medium containing 0.003% 1-phenyl-2-thiourea (PTU) and various concentrations of the tested drugs. The embryos were kept at 28.5 °C and analyzed for survival and gross developmental phenotypes under a stereomicroscope.

### Labelling of MelJuSo cells

MelJuSo cells, were labeled with 1,1′ -dioctadecyl-3,3,3′3′-tetramethylindocarbo-cyanine (DiI) as previously described^72^. Briefly, 70–80% confluent MelJuSo cells were washed with DPBS, covered with DiI at a final concentration of 4 µg/mL and incubated for 30 min at 37 °C. After labeling, cells were detached by pipetting, washed twice in DPBS and kept on ice prior to implantation in zebrafish embryos.

### Zebrafish tumor xenograft-metastasis assay

Transgenic Tg(fli1:EGFP)^y1^ zebrafish embryos^73^ were obtained by natural breeding of adult fish (ZIRC, Eugene, OR USA) and raised in E3-medium supplemented with PTU. MelJuSo cells were resuspended at approximately 10^8^ cells per mL in cell growth medium and approximately 400 cells in 4 nL were implanted in the perivitelline space through sharp glass needles (world precision instruments, pulled in a PC-10 needle puller, Narishige, Tokyo Japan) using a microinjection setup (MINJ-D, TriTech Research, Los Angeles, CA USA). Following injection, embryos were sorted for specific implantation of tumor cells in the perivitteline space and absence of cells in circulation under a fluorescent microscope (SMZ1500, Nikon, Tokyo Japan) and placed in E3 embryo medium containing 0.2 mM PTU. Three days following tumor implantation, the embryos were anesthetized in MS-222 (0.04%, Sigma-Aldrich, St. Lous, MO USA), and primary tumor sizes as well as the extent of local and peripheral, hematologous dissemination/metastasis of tumor cells were visualized under the fluorescent microscope. Results are shown as the average ± standard error of the mean of tumor volumes or number of cells present posterior to the anal opening. All animal experiments were approved by Linköpings Djurförsöksetiska Nämnd and all experiments were performed in accordance with relevant guidelines and regulations.

### Statistical analysis

Student’s t-test, Mann-Whitney U-test and matched pairs t-test were performed using Prism software for Apple computers.

## Supplementary information

Supplementary information

Suppl Fig 5
