# Author Correction: Cytotoxic unsaturated electrophilic compounds commonly target the ubiquitin proteasome system

**DOI:** 10.1038/s41598-019-53667-4

**Published:** 2019-11-20

**Authors:** Karthik Selvaraju, Arjan Mofers, Paola Pellegrini, Johannes Salomonsson, Alexandra Ahlner, Vivian Morad, Ellin-Kristina Hillert, Belen Espinosa, Elias S. J. Arnér, Lasse Jensen, Jonas Malmström, Maria V. Turkina, Padraig D’Arcy, Michael A. Walters, Maria Sunnerhagen, Stig Linder

**Affiliations:** 1 Department of Medical and Health Sciences, Linköping University, SE-58183 Linköping, Sweden; 2 Department of Physics, Chemistry and Biology, Linköping University, SE-58183 Linköping, Sweden; 3 Department of Oncology-Pathology, Karolinska Institutet, SE-17176 Stockholm, Sweden; 4 Division of Biochemistry, Department of Medical Biochemistry and Biophysics, Karolinska Institutet, SE-17177 Stockholm, Sweden; 5 Recipharm AB, Box CB11303, SE-101 32 Stockholm, Sweden; 6 Department of Clinical and Experimental Medicine SE-58185 Linköping University, Linköping, Sweden; 7 Department of Medicinal Chemistry, Institute for Therapeutics Discovery and Development, University of Minnesota, Minnesota, United States

Correction to: *Scientific Reports* 10.1038/s41598-019-46168-x, published online 08 July 2019

This Article contains an error in Figure 8d, where the incorrect brightfield image is shown for the DMSO control embryo at 0 hours. The correct brightfield image appears below.

**Figure 1:**
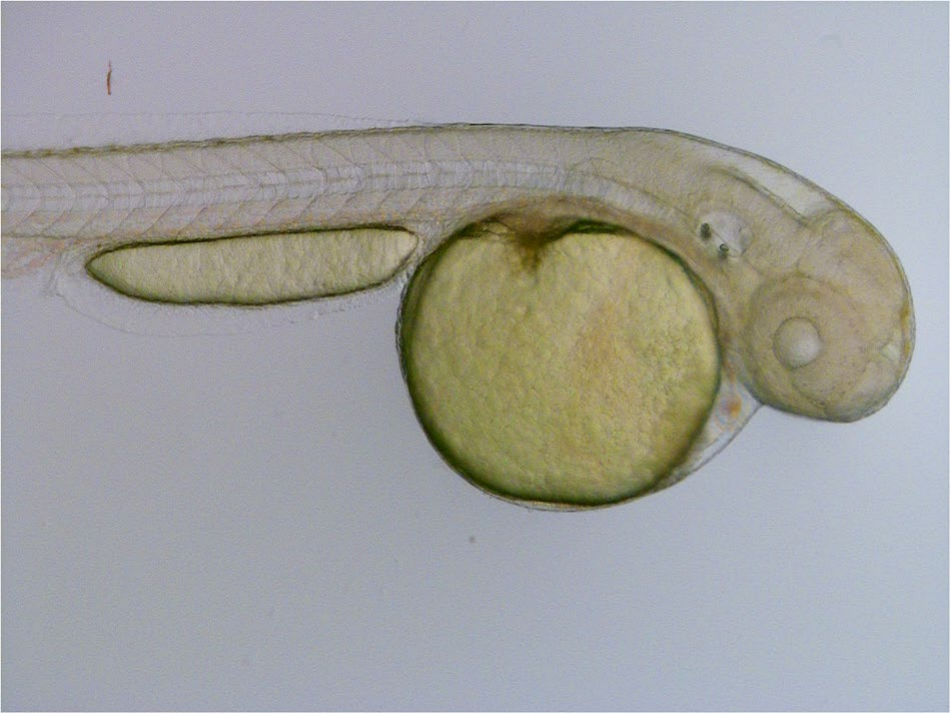
.

The conclusions of the Article are unaffected by these changes.

