# Supplementary material for: Cytotoxic unsaturated electrophilic compounds commonly target the ubiquitin proteasome system
Source: Sci Rep. 2019 Jul 8;9:9841. doi: 10.1038/s41598-019-46168-x (PMC6614553; doi:10.1038/s41598-019-46168-x)
Supplement: Supplementary file 1 — Supplementary information [file 41598_2019_46168_MOESM1_ESM.pdf]

Cytotoxic unsaturated electrophilic compounds commonly target the ubiquitin proteasome system

Karthik Selvaraju[1#], Arjan Mofers[1#], Paola Pellegrini[1], Johannes Salomonsson[2], Alexandra Ahlner[2], Vivian Morad[2], Ellin-Kristina Hillert[3], Belen Espinosa[4], Elias S.J. Arnér[4], Lasse Jensen[1], Jonas Malmström[5], Maria V. Turkina[6], Padraig D'Arcy[1], Michael A. Walters[7], Maria Sunnerhagen[2], and Stig Linder[1, 3]*

Supplementary Table 1

In silico docking of hit compounds to the catalytic domain of USP14.

a) Scored properties of USP14 pockets identified by ICM-Pro, provided by Molsoft. A DLID score >0.5 is considered druggable. Pocket 1 was chosen for docking.

Pocket	Volume	Area	Hydrophobicity	Buriedness	DLID	Radius	Nonsphericity
1	339.7	279.4	0,67	0.90	0.67	4.33	1.19
2	252.3	258.1	0.67	0.83	0.17	3.92	1.34
3	208.7	209.5	0.37	0.76	−0.91	3.68	1.23
4	132.9	198.7	0.35	0.54	−2.15	3.17	1.58
5	170.7	195.3	0.54	0.79	−0.57	3.44	1.31
6	137.7	170.9	0.55	0.87	−0.38	3.20	1.32
7	142.8	158.7	0.55	0.77	−0.77	3.24	1.20
8	141.8	165.6	0.69	0.95	0.27	3.23	1.26
9	106.1	128.6	0.56	0.81	−0.79	2.94	1.19
10	103.3	132.0	0.65	0.78	−0.74	2.91	1.24
11	114.0	121.7	0.49	0.80	−0.96	3.01	1.07

b) Docking score of assayed compounds. A score of -32 or lower is considered good.

Hit compound	Score
CB360	- 19.55
CB997	−19.66
CB686	−19.12
CB688	−24.96
CB113	−22.55
CB729	−19.37
CB742	−22.00
CB916	−20.85
CB826	−22.14
CB383	−39.30

Supplementary Figure 1a

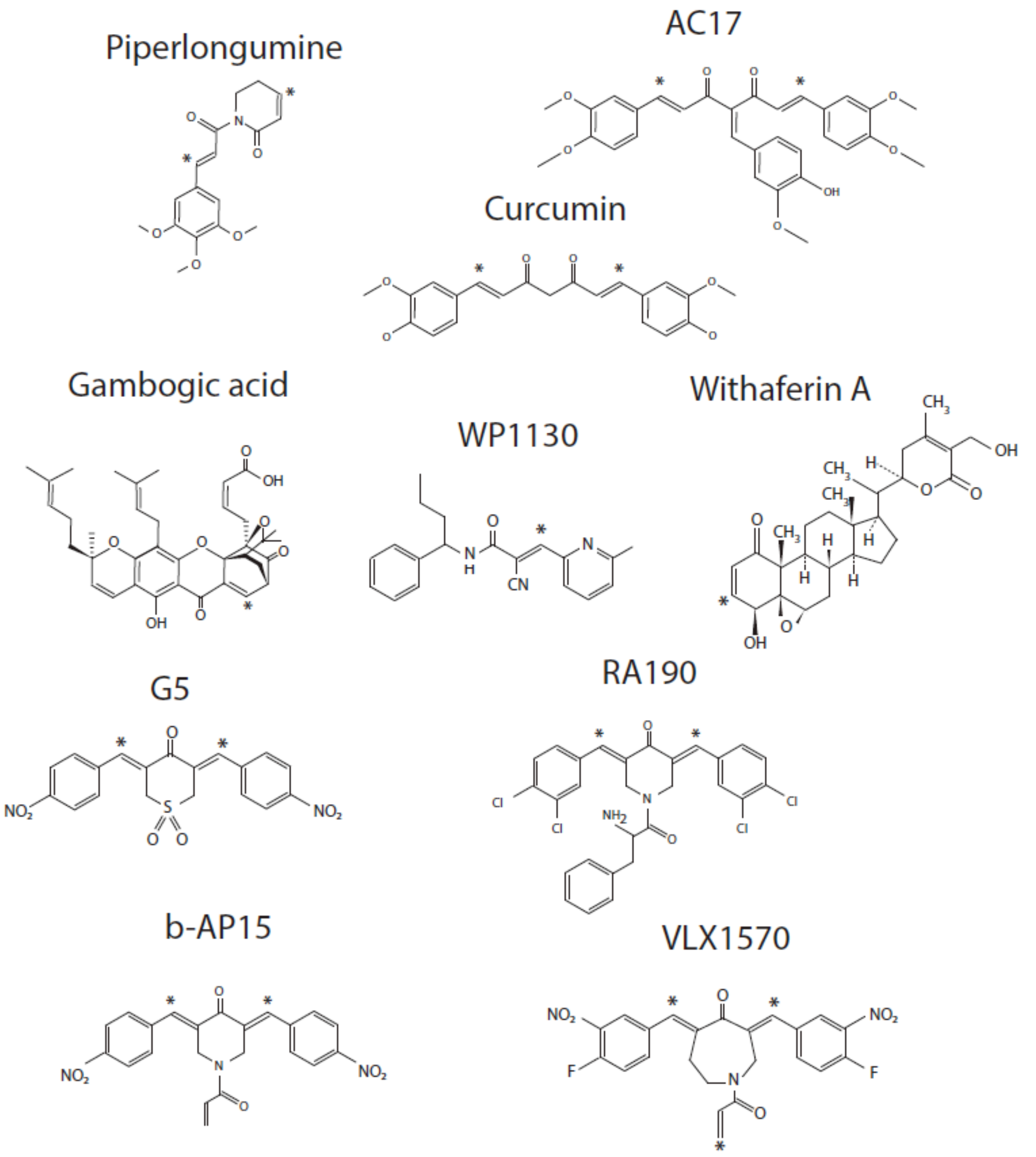

Supplementary Fig 1a. Shown are compounds described as UPS inhibitors relevant to the discussion. Piperlongumine (PMID 23318177, 23772801), curcumin (PMID 23834173, 147018837), gambogic acid (PMID 23260670, 23179339), withaferin A (PMID 25845640), b-AP15 (PMID), G5 (PMID 16982768), VLX1570 (PMID 25854145), WP1130 (21045142), RA190 (PMID 24332045). Curcumin, withaferin A, gambogic acid and piperlongumin are natural products. β-carbons are indicated with asterixes.

Supplementary Figure 1b

7407997 (CB997)

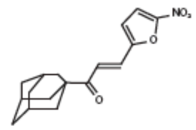

7214729 (CB729)

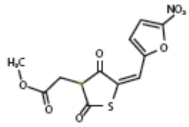

5215686 (CB686)

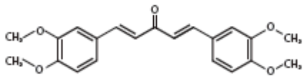

5522742 (CB742)

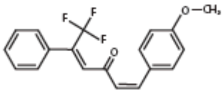

5186383 (CB383)

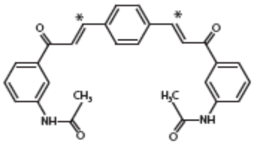

5276688 (CB688)

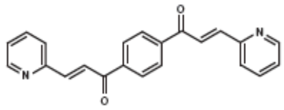

6237916 (CB916)

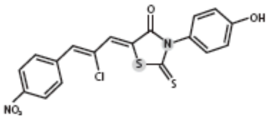

6943113 (CB113)

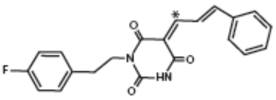

6556826 (CB826)

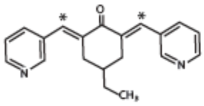

7377360 (CB360)

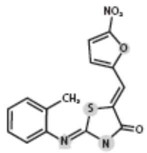

7972237

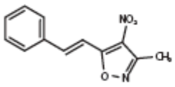

5213883

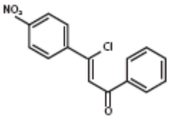

5240652

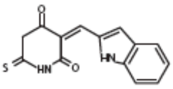

7670140

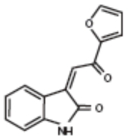

5351900

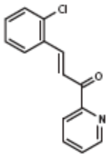

5221596

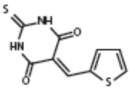

5235209

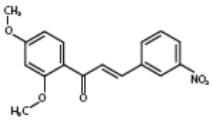

7137104

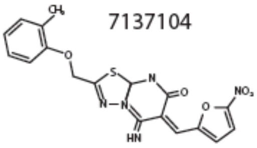

5550131

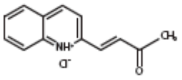

7121239

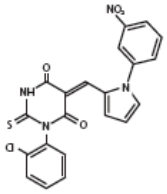

6290394

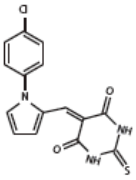

5572643

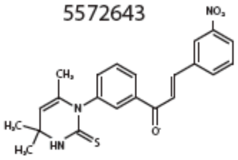

5552328

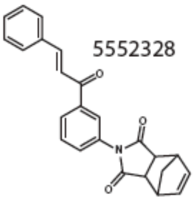

5477925

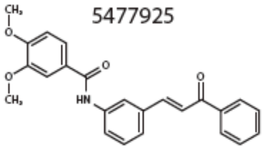

6288863

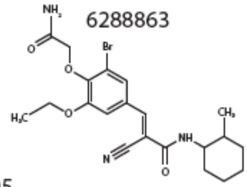

6434784

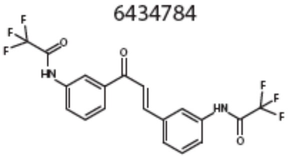

5710114

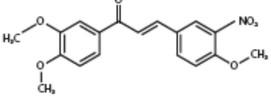

5916305

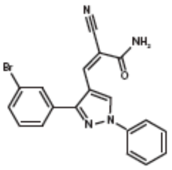

Supplementary Fig. 1b. Structures of the 28 UPS inhibitors identified by screening.
ChemBridge numbers are shown for each compound. The upper 10 compounds were studied in detail.

Supplementary Figure 1c

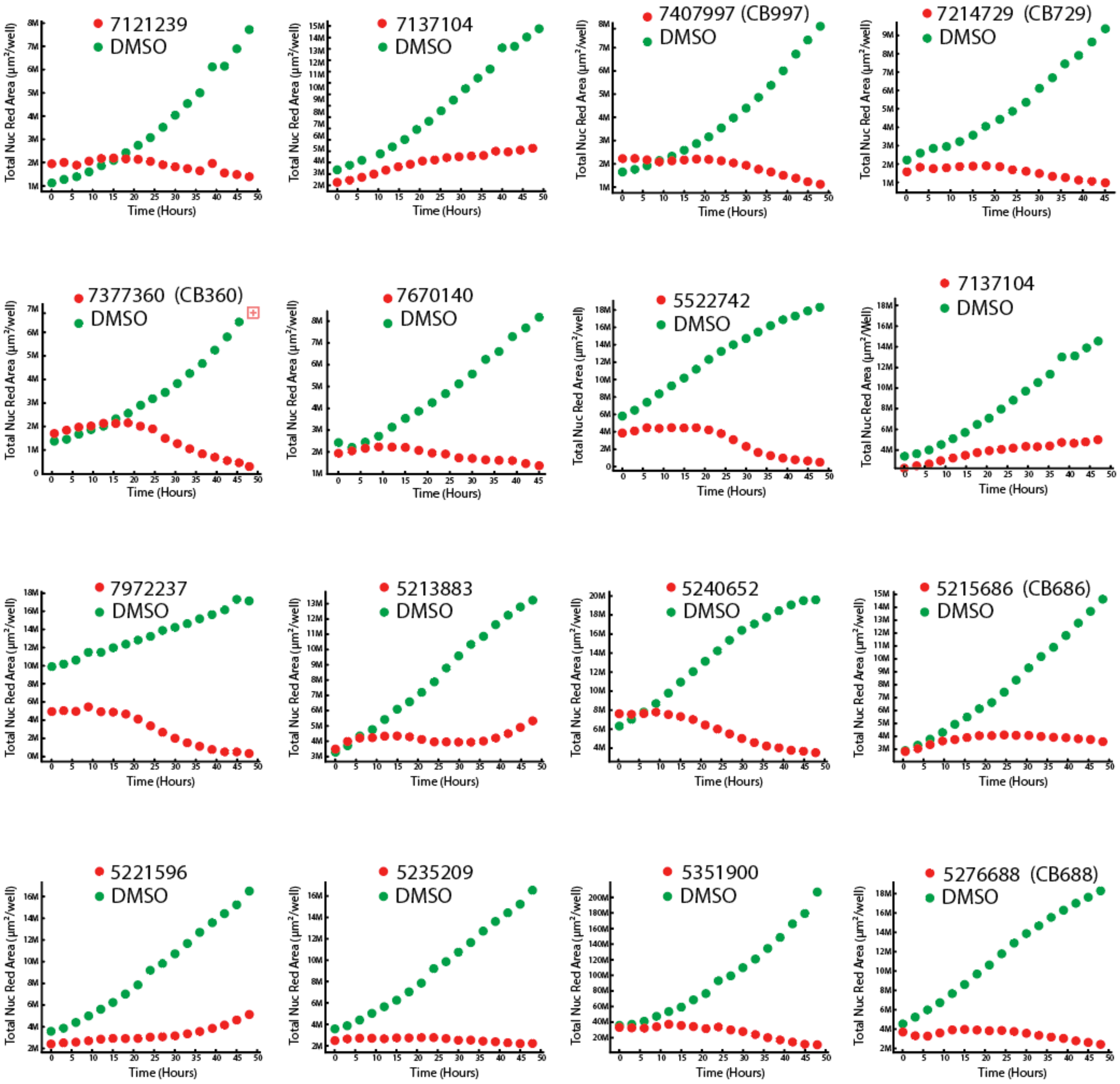

Supplementary Fig 1c. Cell proliferation in the presence of 5 μM of enone compounds. MelJuSo cells were exposed to the the indicated compounds and cell numbers followed in an IncuCyte instrument (as Nuclear Red signals).

Supplementary Figure 1d

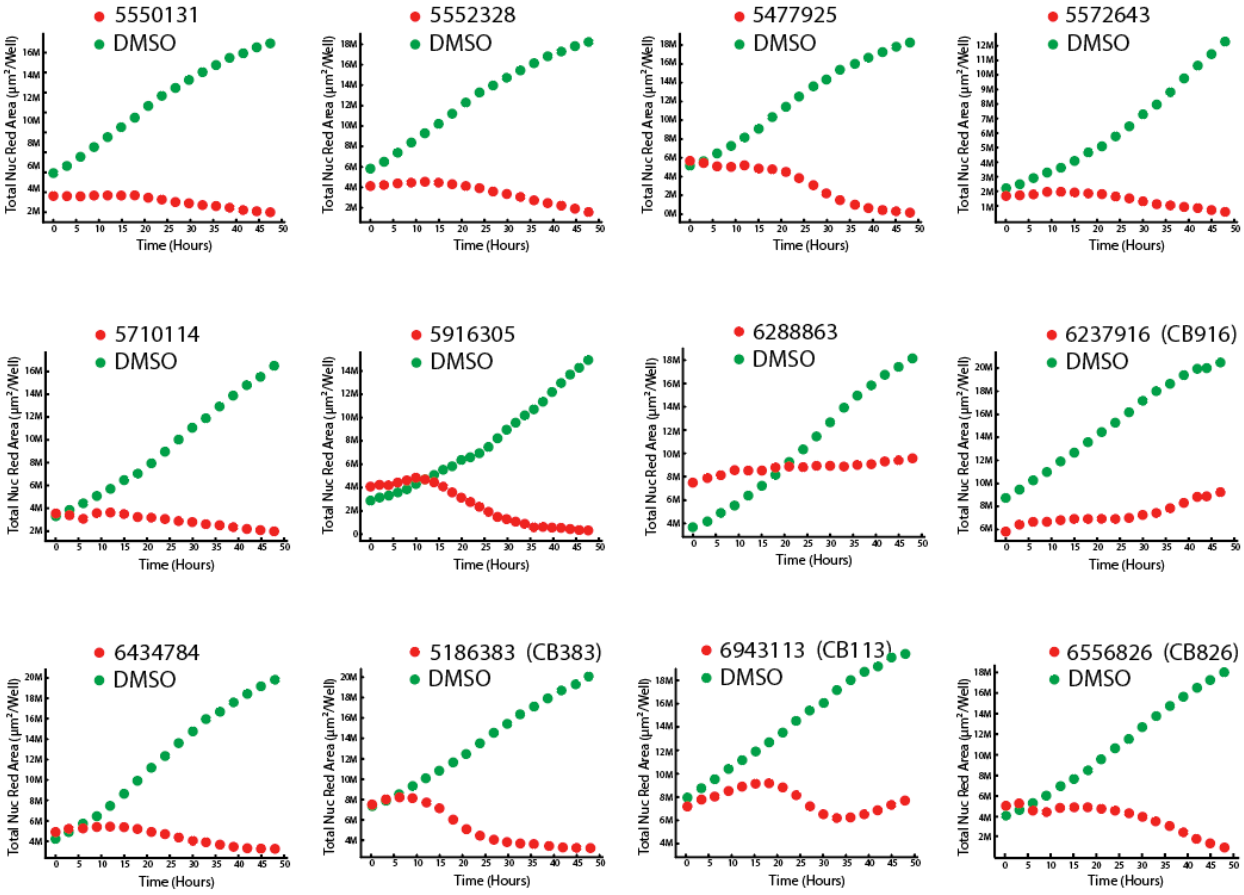

Supplementary Fig 1d. Cell proliferation in the presence of 5 µM of hit compounds. MelJuSo cells were exposed to the the indicated compounds and cell numbers followed in an IncuCyte instrument (as Nuclear Red signals).

Supplementary Figure 2

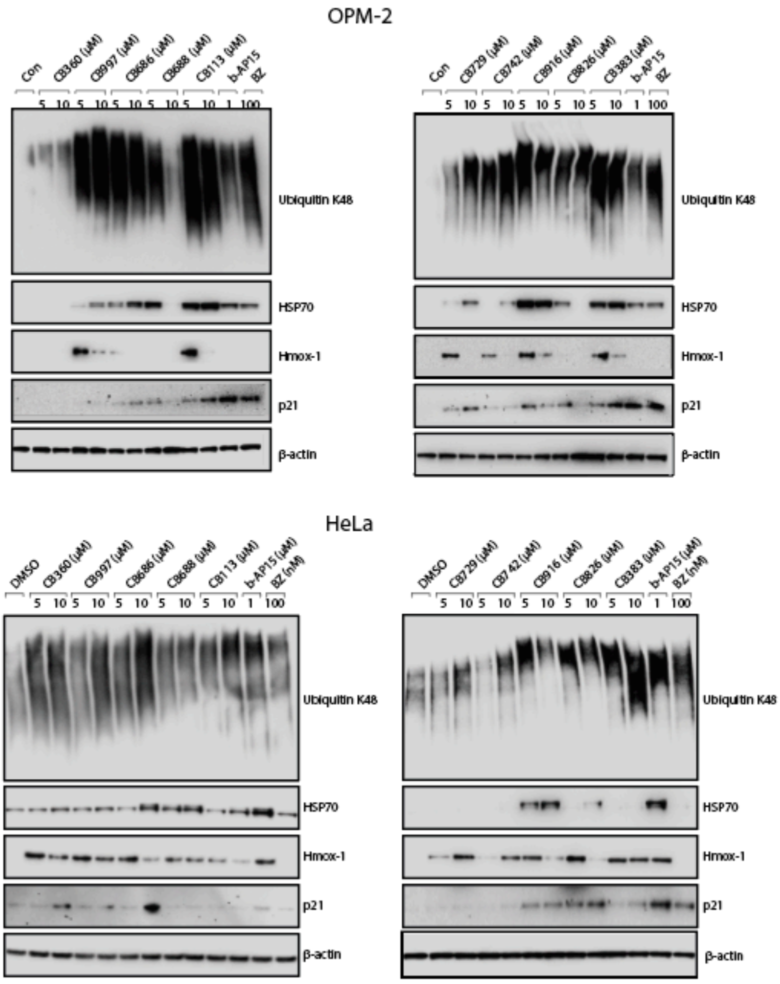

Supplementary Fig 2a. Analysis of the response to hit compounds in OPM-2 myeloma cells and HeLa cells. Cells were exposed to the indicated compounds for 6 hours and subjected to immunoblotting.

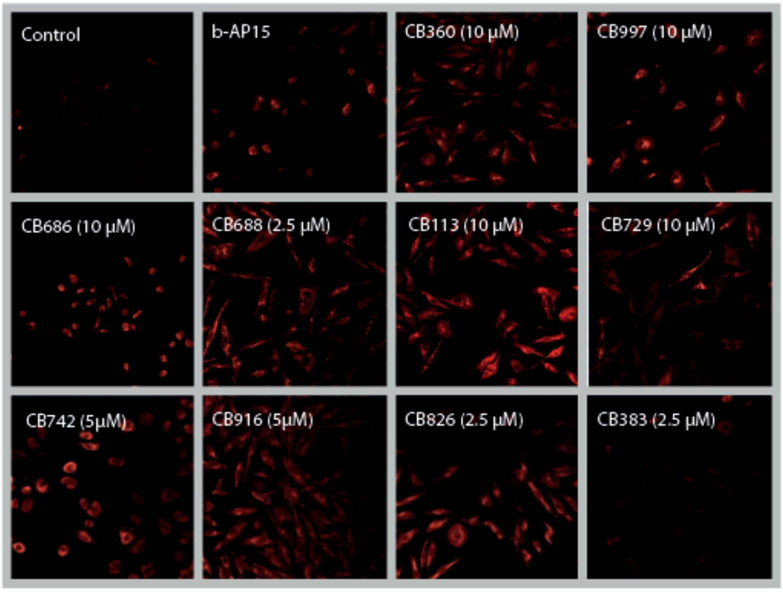

Supplementary Fig 2b. Accumulation of K48-linked polyubiquitinated proteins in HCT116 cells. Cells were exposed to the respective compounds at the indicated concentrations, fixed and subjected to immunohistochemistry.

Supplementary Figure 2c

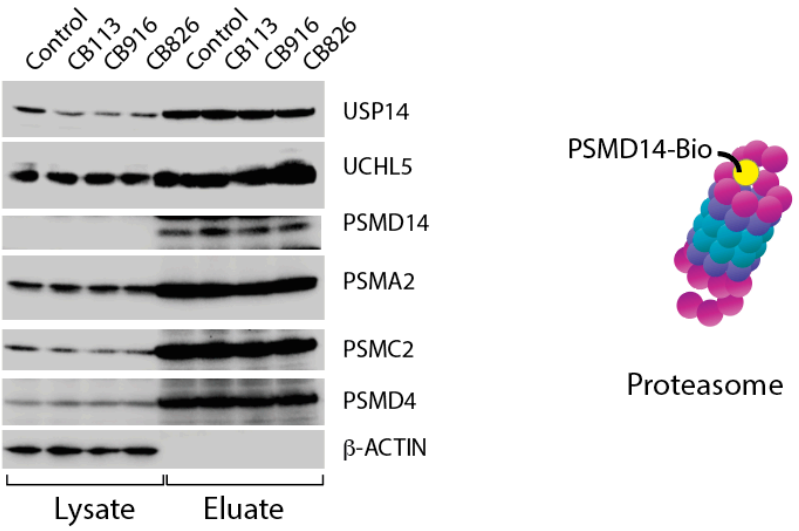

Supplementary Fig 2c. Analysis of proteasome structure using ProteaSelect 293 cells (Wang et al., Biochemistry 46, 3553-3565). Cells were exposed to the indicated compounds (5 μM) and extracts were prepared. Lysates were either analysed directly by immunoblotting or processed for proteasome purification using the Rpn11-biotin tag. Note the purification of both 19S and 20S proteasome subunits, showing integrity of the proteasome during conditions of drug exposure.

Supplementary Figure 3

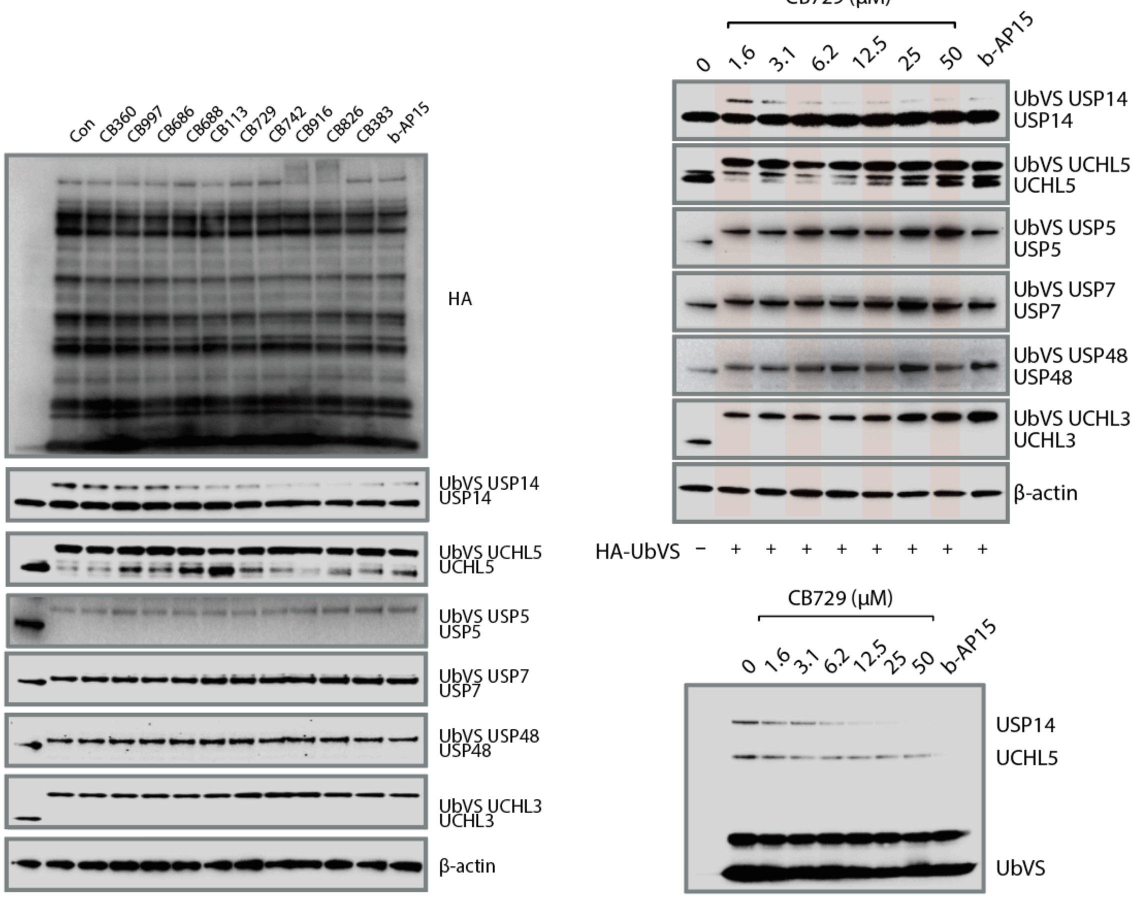

(left) OPM-2 extracts were treated with 50 μM of the indicated compounds followed by labeling with HA-UbVS. Western blots were probed with the indicated antibodies.

(right) Dose response to the nitrofuran compound CB729. OPM-2 cells extracts were exposed to different concentrations of the drug and labeled with HA-UbVS (top panel); 19S proteasomes were exposed to different concentrations of CB729 followed by labeling with HA-UbVS (lower panel). Note that CB729, which showed the highest degreee of electrophilicity in the hit set (Fig. 4d), still shows evidence of selectivity for USP14.

Supplementary Fig. 4a, b

a

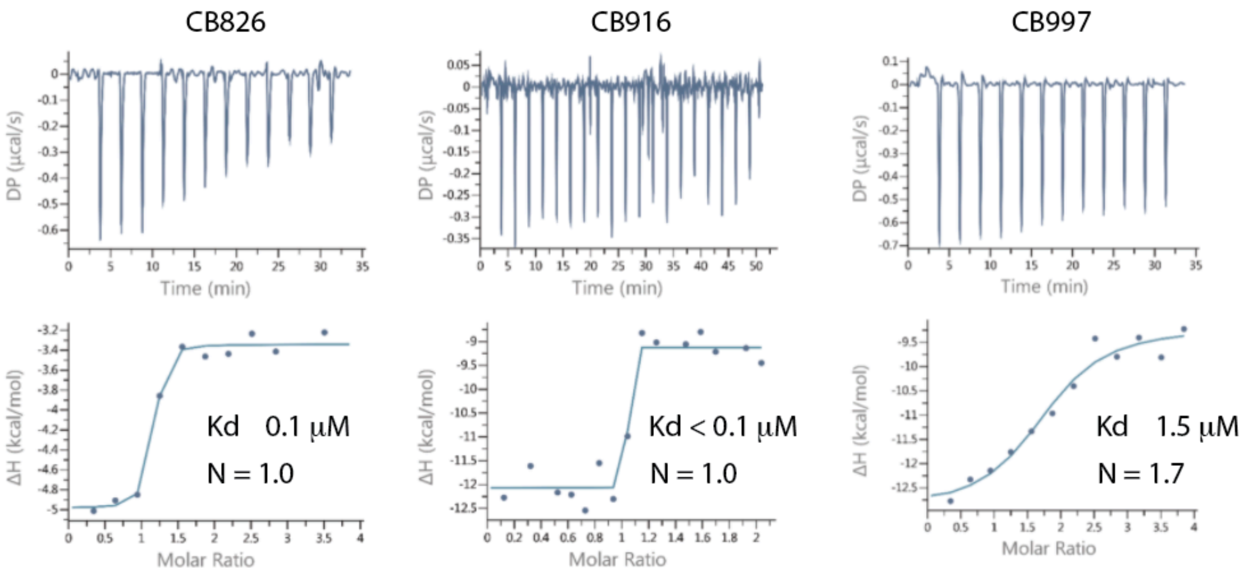

b

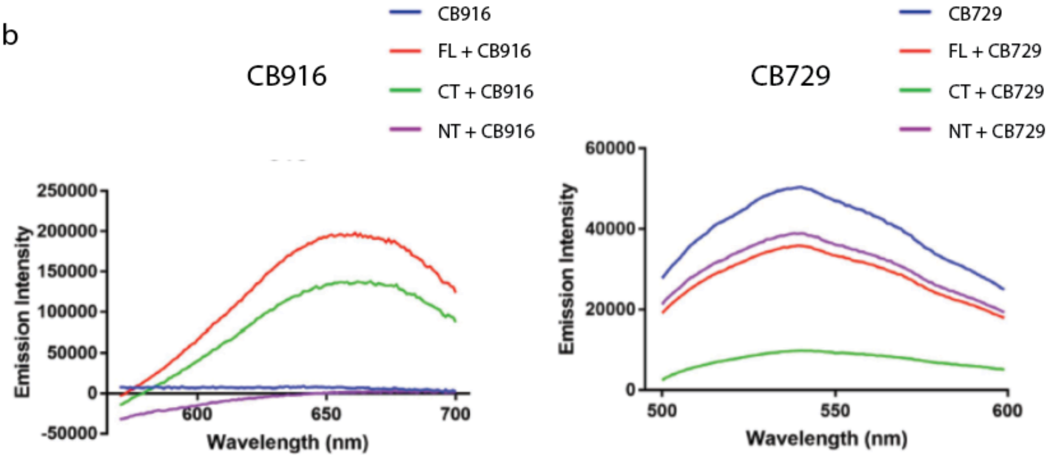

a. ITC data for USP14 interaction with three hit compounds at 25°C. Titration of CB826 (500 µM, 13 automatic injection), CB916 (100 µM, 20 automatic injection), and CB997 (100 µM, 13 automatic injection) to USP14 (25 µM/10 µM/10µM). The upper panel shows the heat flow for each injection (µcal/s) as a function of time (min), while the bottom panel shows the integrated heats of each injection. The fitted dissociation constants (KD) and number of binding sites (N) were calculated using Malvern MicroCal Software for PEAQ-ITC Analysis.
b. Spectral scan of hit compounds CB916 (left) and CB729 (right) alone (blue) and with USP14-FL (red), USP14-CD (green) and USP14-Ubl (purple)

Supplementary Fig. 4c

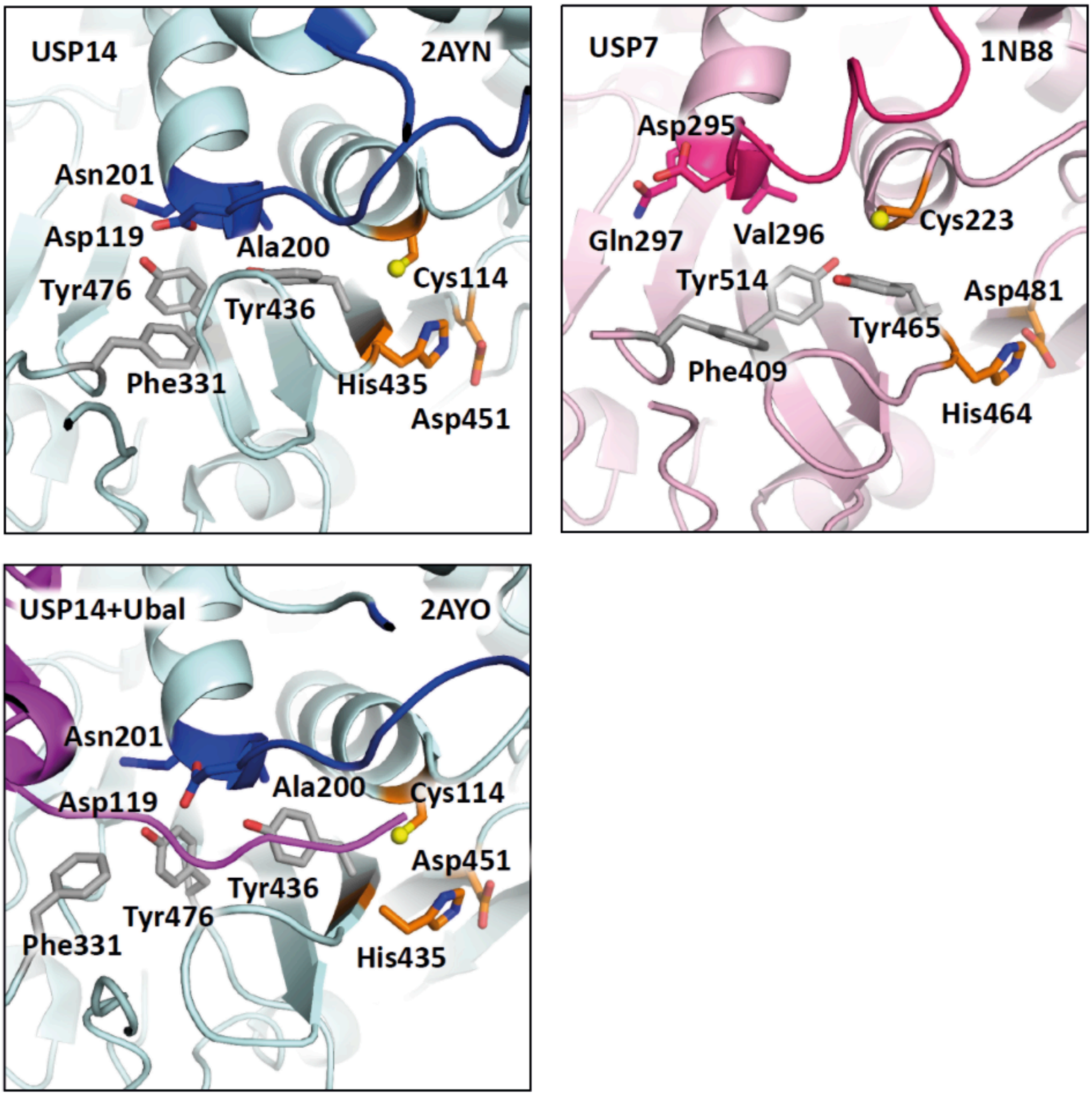

Structural similarities in the thumb-palm clefts of DUBs USP14 and USP7.
(upper left) USP14 , (upper right) USP7 apo, (lower left) USP14 with c-terminal tail of anchored Ub.

Annotated residues are involved in inhibitor binding in USP7 (Turnbull et al., Nature 2017), with structurally homologous residues indicated in USP14. D) Sequence alignment of the switching loops in USP7 and USP14 shows limited sequence similarities; residues indicated in sticks in A-C and involved in USP7 inhibitor binding are boldfaced.

Supplementary Fig. 6.

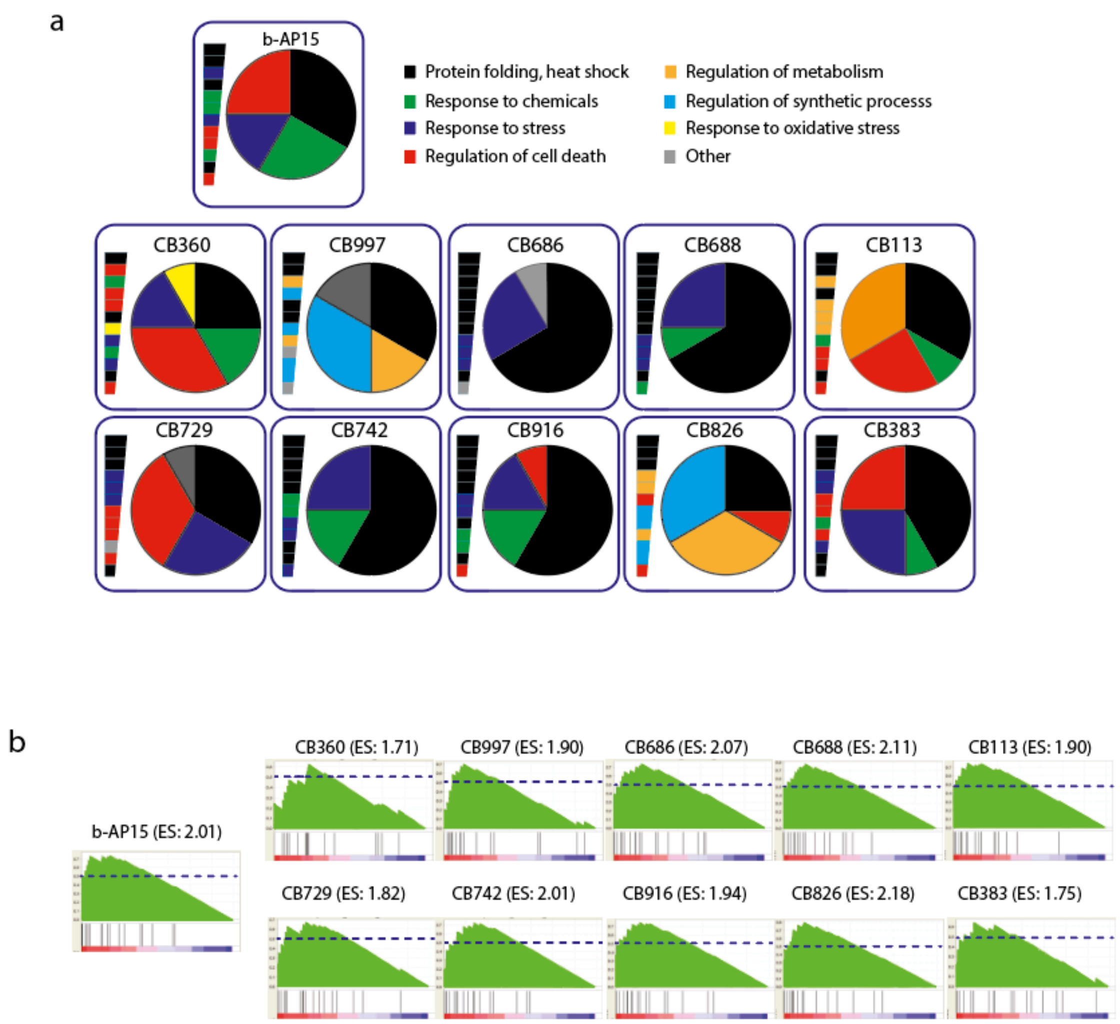

(a) Gene ontology analyses of the responses to the different compounds.
The 12 most commonly found gene ontologies for each compound were scored and ranked (bar to the left) and visualized as pie charts (right); (b) Gene enrichment analysis (GSEA) for genes involved in protein folding. Note the similar enrichment scores for the different compounds.

Figure 2: western blot images

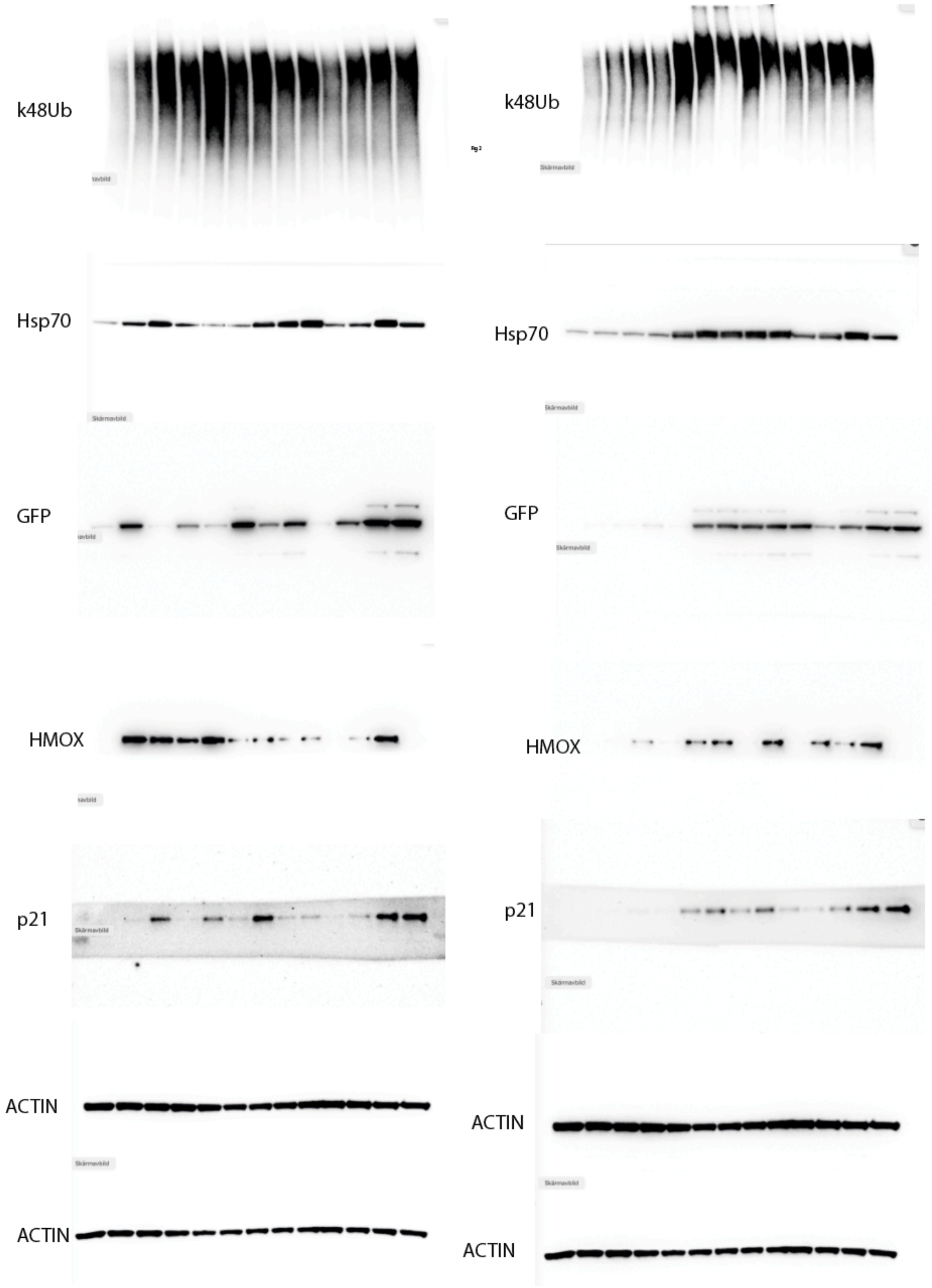

Fig 4c: western blot images

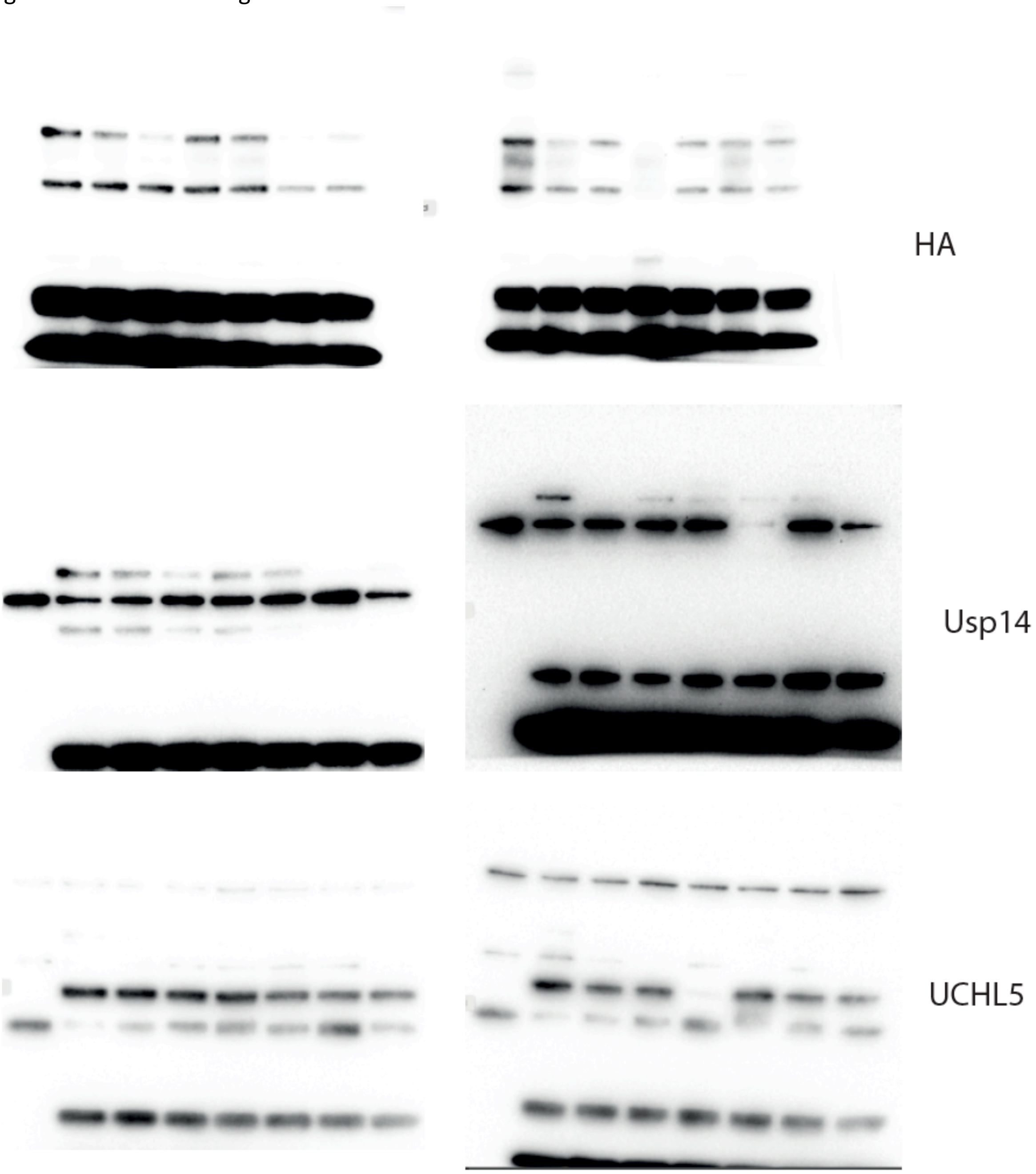

Fig 4e: western blot images

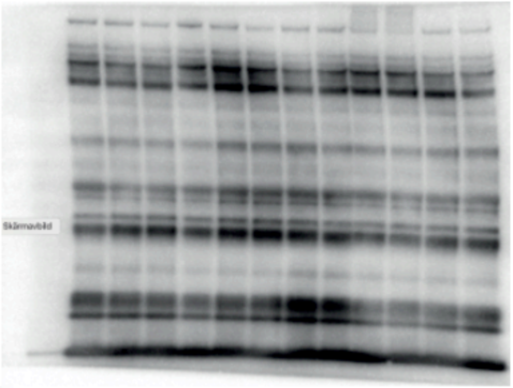

HA antibody

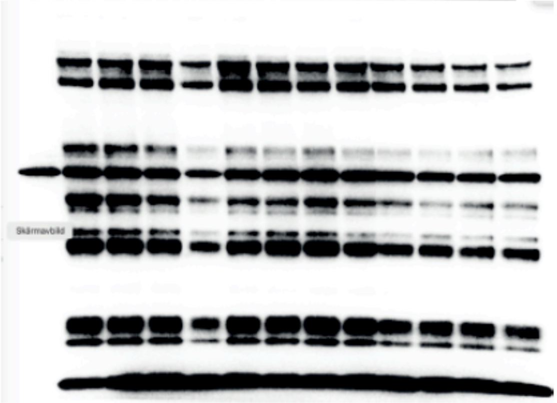

USP14 antibody

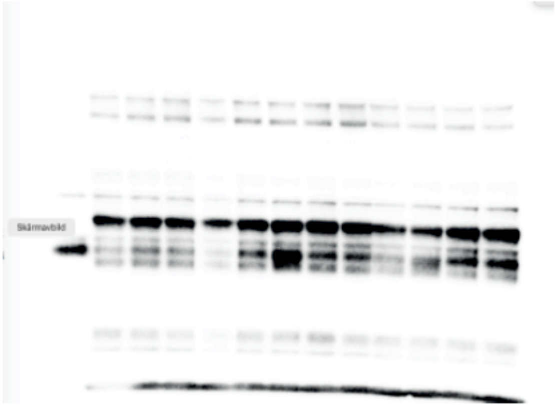

UCHL5 antibody

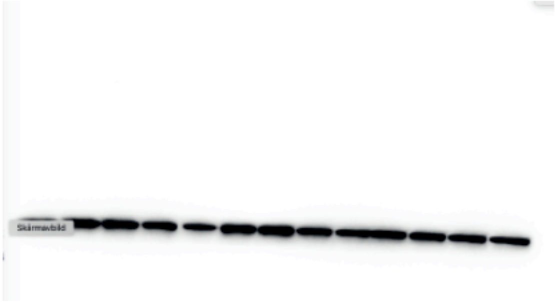

β-actin antibody

Fig 7a

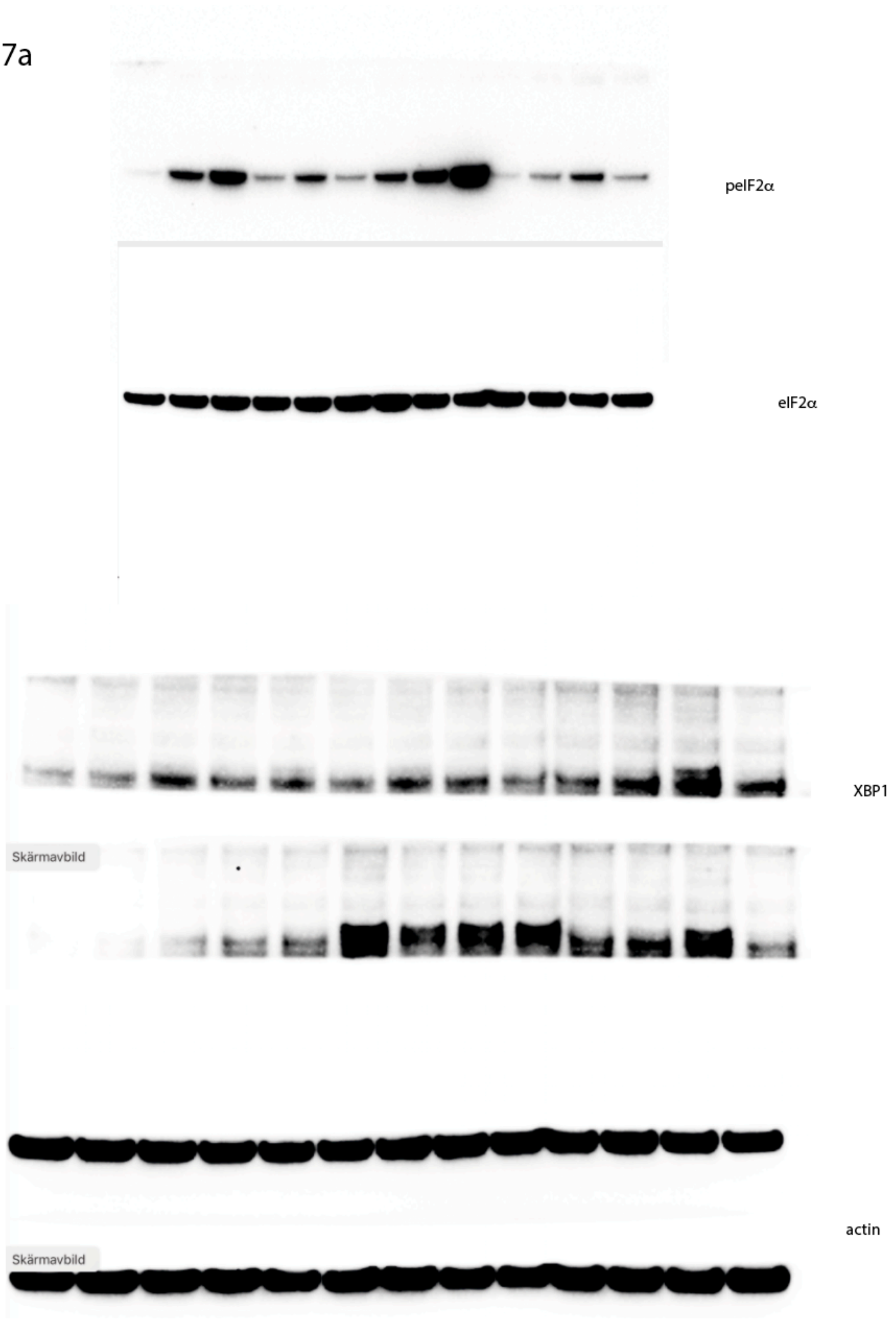

Fig 7a OPM2

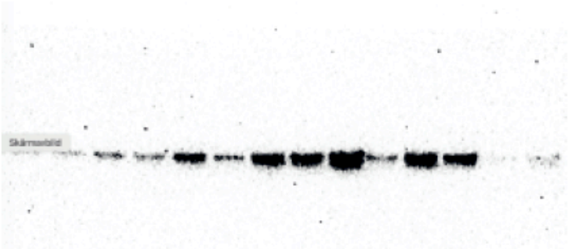

p-eIF2a

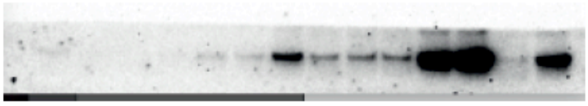

XBP1 (filter cut to allow for multiple antibodies)

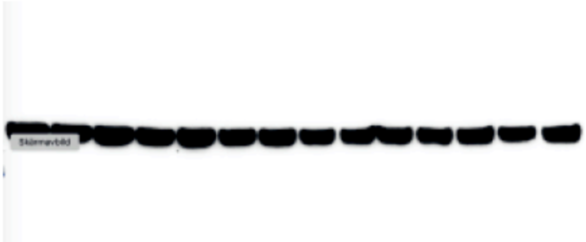

eIF2a

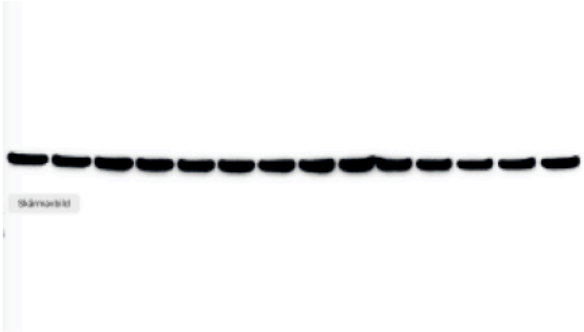

ACTIN

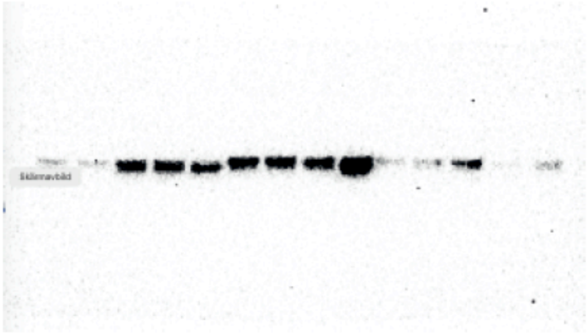

p-eIF2a

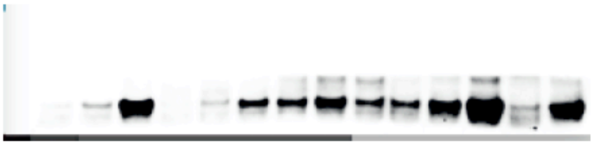

XBP1 (filter cut to allow for multiple antibodies)

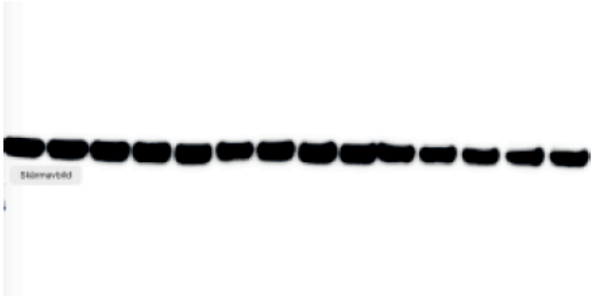

eIF2a

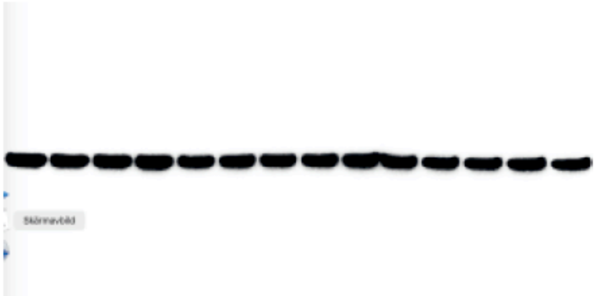

ACTIN

Fig 7b

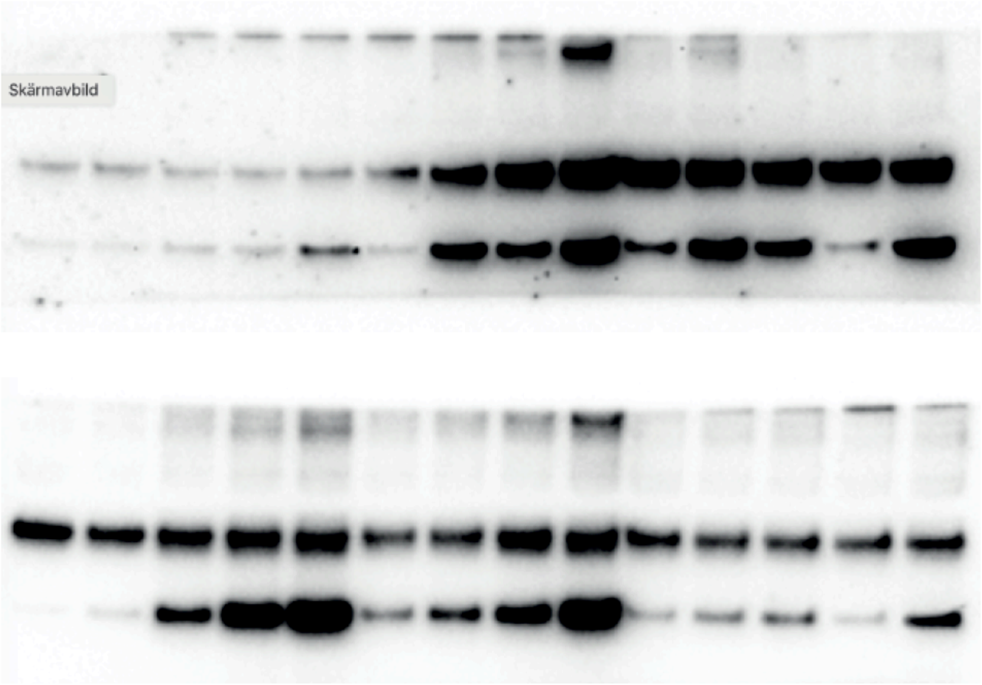

casp-3

casp-3
